# GSH Levels Serve As a Biological Redox Switch Regulating Sulforaphane-Induced Cell Fate in Human Lens Cells

**DOI:** 10.1167/iovs.62.15.2

**Published:** 2021-12-02

**Authors:** Thao Phuong Ngoc Huynh, Richard P. Bowater, Federico Bernuzzi, Shikha Saha, I. Michael Wormstone

**Affiliations:** 1 School of Biological Sciences, University of East Anglia, Norwich, United Kingdom; 2 Quadram Institute, Norwich Research Park, Norwich, United Kingdom

**Keywords:** lens, posterior capsule opacification, sulforaphane, glutathione, oxidative stress

## Abstract

**Purpose:**

Sulforaphane (SFN) is a therapeutic phytochemical agent for many health conditions. SFN-induced cytotoxicity is shown to have promise in preventing posterior capsule opacification (PCO). In the current study, we aimed to elucidate key processes and mechanisms linking SFN treatment to lens cell death.

**Methods:**

The human lens epithelial cell line FHL124 and central anterior epithelium were used as experimental models. Cell death was assessed by microscopic observation and cell damage/viability assays. Gene or protein levels were assessed by TaqMan RT-PCR or immunoblotting. Mitochondrial networks and DNA damage were assessed by immunofluorescence. Mitochondrial membrane potential, activating transcription factor 6 (ATF6) activity, ratio of reduced glutathione (GSH) to oxidized glutathione (GSSG), and glutathione reductase (GR) activity were measured using different light reporter assays. SFN metabolites were analyzed by LC-MS/MS.

**Results:**

Treatment with *N*-acetylcysteine (NAC), a reactive oxygen species scavenger, prevented SFN-induced cell death in both models. NAC also significantly protected FHL124 cells from SFN-induced mitochondrial dysfunctions, endoplasmic reticulum stress (ERS), DNA damage and autophagy. SFN significantly depleted GSH, the major antioxidant in the eye, and reduced GR activity, despite doubling its protein levels. The most abundant SFN conjugate detected in lens cells following SFN application was SFN–GSH. The addition of GSH protected lens cells from all SFN-induced cellular events.

**Conclusions:**

SFN depletes GSH levels in lens cells through conjugation and inhibition of GR activity. This leads to increased reactive oxygen species and oxidative stress that trigger mitochondrial dysfunction, ERS, autophagy, and DNA damage, leading to cell death. In summary, the work presented provides a mechanistic understanding to support the therapeutic application of SFN for PCO and other disorders.

The use of phytochemicals for therapeutic purposes is a growing area of medicine.^1^^–^^3^ Sulforaphane (SFN) is one such molecule. SFN is an isothiocyanate derived from glucosinolate glucoraphanin found in cruciferous vegetables, such as broccoli and kale.^4^ This hormetic chemical displays contrasting properties depending on concentration and induces myriad cellular events.^5^^,^^6^ At low doses, SFN can trigger the activation of phase-II detoxification enzymes, increase antioxidant defense, decrease inflammatory responses, and promote cell survival via the nuclear factor erythroid 2–related factor 2 (Nrf2) pathway. High levels of SFN block cell proliferation and induce cell death. Due to its multifaceted in vivo and in vitro effects, SFN has selectively been studied to treat cancer, metabolic disorders, or inflammatory conditions.^7^^–^^10^

Recently, SFN has shown potential for treating a lens disorder called posterior capsular opacification (PCO).^11^ Cataract, a clouding of the lens, is a leading cause of blindness in the world; surgery is currently the only effective treatment. Unfortunately, the post-surgery complication PCO can cause approximately 10% to 20% of patients to lose their vision again.^12^ In this condition, remaining lens cells proliferate, invade the previously cell-free regions, and eventually disrupt the visual axis. An Nd:YAG laser can treat PCO, but it is costly and has associated risks. It is accepted that prevention of PCO is preferable, and to this end efforts have been directed toward improving the design of intraocular lenses,^13^^–^^15^ routinely implanted during cataract surgery, to suppress PCO progression. Pharmacological approaches may also yield benefits, but currently none is in clinical use.^12^^,^^16^ However, SFN has shown promise. Liu et al.^11^ demonstrated that when applied to human capsular bags, a model for PCO, high supranutritional SFN concentrations caused cell death to remaining cells and prevented opacification, suggesting SFN as a potential agent to manage PCO. To utilize all therapeutic potentials of SFN for PCO and other disorders, first and foremost it is important to understand the cellular and molecular mechanisms that mediate SFN-induced cytotoxicity.

Multiple mechanisms potentially govern SFN-induced cytotoxicity.^6^ Aspects proposed to play a role in this process include a vicious oxidative cycle, mitochondrial dysfunction, endoplasmic reticulum stress (ERS), and glutathione (GSH) depletion.^6^^,^^17^^,^^18^ However, a complete interplay between events has not been well linked in either cancer or non-cancer models. A study linking multiple processes in a non-cancer human experimental system is required to address gaps in our knowledge and provide important mechanistic information to understand the pharmacological actions of SFN. The lens affords a model system that is well placed to tease out key processes and mechanisms linking SFN treatment to cell death. The lens epithelium is a distinct population of cells that naturally grows in isolation and in the absence of vasculature. These traits, along with well-defined molecular characteristics, render lens cell and tissue cultures a tractable experimental system for the study of SFN. In the present study, we significantly expanded on the previous findings of Liu et al.^11^ to demonstrate that SFN-induced depletion of GSH triggers oxidative stress leading to mitochondrial dysfunction, ERS, DNA damage, autophagy, and ultimately cell death of human lens cells. The findings of the study, therefore, provide valuable mechanistic information to support the therapeutic application of SFN for the treatment of human disease and the importance of redox status in SFN-mediated cell death.

## Materials and Methods

### Reagents

R-sulforaphane (Enzo Life Sciences, Farmingdale, NY, USA) was dissolved in sterile dimethylsulfoxide (DMSO) to achieve a stock concentration of 50 mM. *N*-acetyl-l-cysteine (Sigma-Aldrich, St. Louis, MO, USA) was dissolved in an appropriate culture medium and filtered to reach a stock concentration of 100 mM. Sodium pyruvate (Sigma-Aldrich) was dissolved in sterile double-distilled water to achieve a stock concentration of 1 M. Then, reduced l-glutathione (Sigma-Aldrich) was dissolved in PBS at pH 6.5 and filtered to achieve a stock concentration of 100 mM.

### Cell Culture

Fetal human lens-124 (FHL124) is a non-virally transformed cell line generated from human capsule–epithelial explants and shares a 99.5% homology with the native lens epithelium in the transcript profile.^19^ FHL124 cells were routinely cultured at 35°C with 5% CO_2_, 95% air in Eagle's Minimum Essential Medium (EMEM) (Gibco, Waltham, MA, USA) supplemented with 5% v/v fetal bovine serum (FBS; Gibco) and 50 µg/mL gentamycin (Sigma-Aldrich). FHL124 cells (passage numbers 12–24) were seeded onto 35-mm tissue culture dishes (Corning, Corning, NY, USA) for protein and RNA extraction; 18 × 18-mm glass coverslips contained within 35-mm culture dishes for immunocytochemistry, 100-mm tissue culture dishes (Corning) for metabolite extraction, or 96-well plates for several cell behavioral assays (Nalge Nunc International, Rochester, NY, USA, if not stated otherwise). Cells were maintained in EMEM with 5% FBS and replaced with EMEM without FBS 24 hours before the start of experimental conditions.

### Human Lens Epithelium

Human donor eyes with the cornea removed (ages 47–89 years) were obtained from the NHSBT Filton Eye Bank (UK) with informed consent and used in accordance with the tenets of the Declaration of Helsinki. Human eyes were enucleated within 24 hours of death. Approval for the study and experimental protocols (NREC 04/Q0102/57–IRAS D 293806) was granted by a national research ethics committee under the Health Research Authority (UK). Using an insulin needle, the anterior capsule was breached approximately 3 mm from the equator, and an incision was made from that point to the center of the capsule. A continuous curvilinear capsulorhexis was created by tugging the flap created by this incision with surgical forceps. The capsulorhexis was then secured onto a non-treated, 30-mm, triple-vent petri dish (Thermo Fisher Scientific, Waltham, MA, USA) entomological pins and maintained in EMEM supplemented with 5% FBS and 50 µg/mL gentamicin. The tissues were changed into EMEM without serum 24 hours before experiments.

### Cell Viability Assay

FHL124 cells were seeded onto clear 96-well plates at a density of 2500 cells/well, and cell viability was measured using the CellTiter-Glo Luminescent Assay (Promega, Madison, WI, USA) following the manufacturer's instructions. The working solution was transferred to a white, opaque 96-well plate compatible with luminescence reading, and the plate was immediately analyzed for luminescence on a FLUOstar Omega microplate reader (BMG LabTech, Ortenberg, Germany).

### Cytotoxicity Assay

A lactate dehydrogenase kit (Roche, Basel, Switzerland) was used to assess cytotoxicity. At the end of the experiments, for FHL124 cells 50 µL of the culture medium of FHL124 cells seeded at a density of 50,000 cells per culture dish was sampled and transferred to a clear 96-well plate already containing 150 µL EMEM per well. For human lens epithelium, 100 µL of culture medium from a whole capsulorhexis stabilized by pins in a culture dish and 100 µL EMEM medium were used instead. A working solution was made following the manufacturer's instruction, and 100 µL of the working solution was then added into each well. The whole plate was then covered in foil and incubated on a shaker at room temperature for 15 minutes before being measured with the FLUOstar Omega microplate reader at 490 nm.

### ATF6 Reporter Assay

FHL124 cells were seeded onto clear 96-well plates at a density of 5000 cells/well, and activating transcription factor 6 (ATF6) activity was measured by the ATF6 report assay as described by Wang et al.^20^

### Quantitative RT-PCR

Total RNA was extracted from FHL124 cells using the ReliaPrep RNA Miniprep System (Promega) following the manufacturer's instructions. RNA was quantified using a NanoDrop spectrophotometer (Thermo Fisher Scientific), and cDNA was generated using a PCR Biosystems kit (PCR Biosystems, London, UK) following the manufacturer's instructions. The cycle threshold (CT) value was used to assess gene expression by the 2^−^^ΔΔCT^ method. Fold amplification was normalized with *18S* as an endogenous control gene using predesigned TaqMan probes and TaqMan PCR master mixes (Applied Biosystems, Waltham, MA, USA):

• *DDIT3* (C/EBP homologous protein; reference sequence NM_001195053; TaqMan primer Hs00358796_g1)
• *ERN1* (serine/threonine–protein kinase/endoribonuclease; reference sequence NM_001433; TaqMan primer Hs00980095_m1)

### Western Blot Analysis

Cell lysates from FHL124 cells and human lens epithelium were prepared using M-PER buffer (Thermo Fisher Scientific) supplemented with phosphatase and protease inhibitors and 0.5-M EDTA (Thermo Fisher Scientific) at 10 µL/mL immediately before use. Total protein content was determined by the BCA Protein Assay (Thermo Fisher Scientific) to enable the loading of equal amounts of protein per sample onto SDS-polyacrylamide gels. Proteins were transferred onto polyvinyl difluoride (PVDF) membranes using a Trans-Blot Turbo Transfer System (Bio-Rad Laboratories, Hercules, CA, USA). PVDF membranes were blocked with PBS containing 3% w/v BSA and 0.1% v/v Tween-20, hybridized with primary antibodies at 4°C overnight, followed by incubation with secondary antibody conjugated with horseradish peroxidase (GE Healthcare, Chicago, IL, USA). Proteins were detected using the Clarity Western ECL Substrate (Bio-Rad Laboratories) and visualized with a ChemiDoc imaging system (Bio-Rad). β-Actin was used as a loading control and for band intensity normalization. The following antibodies were used: anti-phosphor-eIF2α (3398, Cell Signaling Technology, Danvers, MA, USA), anti-XBP1 (ab220783; Abcam, Cambridge, UK), anti-LC3 (L8918; Sigma-Aldrich), and anti-β-actin (4970; Cell Signaling Technology).

### Immunocytochemistry for DNA Damage

FHL124 cells were seeded onto coverslips at a density of 5000 cells/well. After the end of the experimental conditions, coverslips were fixed with 4% v/v formaldehyde (Sigma-Aldrich) in PBS for 20 minutes. Three washes in PBS were performed before blocking for nonspecific binding sites with 3% normal donkey serum in 1% w/v BSA/PBS for 1 hour at 37°C. Anti-γ-H2AX (Cell Signaling Technology) was diluted 1:200 in 1% BSA/PBS and applied to cells for 2 hours at 37°C. The incubation was followed by another three PBS washes. Then, cells were added with the secondary antibody Alexa Fluor 488 conjugated Donkey anti-Rabbit (Thermo Fisher Scientific) at 1:200 dilution in 1% BSA/PBS for 1 hour, protected from light at 37°C in a humidified atmosphere. Coverslips were then counterstained with 4′,6-diamidino-2-phenylindole (DAPI; Sigma-Aldrich) for nucleus staining and Texas Red-X phalloidin (Thermo Fisher Scientific) for actin (if used), both at 1:200 in 1% w/v BSA/PBS for 10 minutes at room temperature. After three washes, samples were mounted on glass microscope slides with Hydromount. Samples were viewed with a Zeiss Axioplan 2ie widefield microscope (Carl Zeiss Microscopy, Jena, Germany). Image quantification was performed using ImageJ 1.45 (National Institutes of Health, Bethesda, MD, USA).

### Mitochondrial Labeling

To observe mitochondria in fixed cells, FHL124 cells were seeded onto coverslips at a density of 2000 cells and labeled with Invitrogen MitoTracker Red CMXRos (Thermo Fisher Scientific) dissolved in DMSO. This was achieved by incubating the cells with phenol red free EMEM culture media containing 100-nM dye for 15 minutes within the culture incubator at the end of experimental conditions, followed by three washes with prewarmed medium. Cells were then fixed and mounted as described above. Cells were individually imaged with a Zeiss LMS980 confocal microscope with Airyscan 2 or with a Zeiss LSM 510 Meta microscope at 63× with oil. After image acquisition, the size of mitochondrial networks was analyzed using ImageJ 1.45. The analysis procedure was modified from a previously reported protocol.^21^ The workflow is described in Supplementary Figure S1.

### Mitochondrial Membrane Potential

FHL124 cells were seeded onto black-plate, clear-bottom 96-well plates (PerkinElmer, Waltham, MA, USA) at a density of 5000 cells/well. Mitochondrial membrane potential was assessed using the tetramethylrhodamine ethyl ester (TMRE) Mitochondrial Membrane Potential Assay Kit (Abcam) following the manufacturer's instructions. The chosen concentration of TMRE was 400 nM. The signal was measured using the FLUOstar Omega microplate reader at excitation (Ex)/(emission) Em = 549/575 nm every 3 minutes for 15 minutes. Peak signals were chosen for analysis.

### SFN Metabolites Analysis

FHL124 cells were seeded onto 100-mm culture dishes until 95% confluency in 10 mL medium. The culture medium was aspirated, and cells were washed twice with cold 0.9% sodium chloride (Sigma-Aldrich). Cells were then trypsinized with 0.05 % trypsin/EDTA following the laboratory's standard procedure and counted using a hemocytometer. Next, the solutions were collected into 25 mL Sterilin tubes (Thermo Fisher Scientific) and were centrifuged at 1200*g* at room temperature for 10 minutes. The supernatant was removed, and the pellets were resuspended in 10 mL of prewarmed 0.9% sodium chloride, and the solutions were centrifuged again at 1200*g* at room temperature for another 10 minutes. The supernatant was removed, but this time the pellets were resuspended in 0.5 mL of cold 0.3-mM perchloric acid, and the solutions were kept on ice for 10 minutes. After that, the solutions were transferred to 1.5-mL microcentrifuge tubes and centrifuged at 13,000*g* at 4°C for 10 minutes. The supernatant was collected and stored at −80°C for further analysis.

To measure free SFN in culture medium (EMEM) 1 hour following the addition of SFN in the presence or absence of 1-mM *N*-acetylcysteine (NAC) or 1-mM GSH, 1 mL of culture medium was transferred into a 1.5-mL Eppendorf tube containing 100 µL 3-mM perchloric acid (Sigma-Aldrich), and the mixture was stored at −20°C prior to liquid chromatography–mass spectrometry (LC-MS) analysis.

SFN and its conjugates, SFN-cysteine (SFN–Cys), SFN–GSH, SFN–Cys–Gly, and SFN–NAC, were measured in cells using a validated LC-MS mass spectrometer method (LC tandem mass spectrometry [LC-MS/MS]).^22^ A five-point calibration curve was generated by twofold serial dilution of standards (SFN, SFN–Cys, SFN–GSH, SFN–NAC, and SFN–Cys–Gly) in Milli-Q pore water (MilliporeSigma, Burlington, MA, USA) for cells and EMEM media for media. The starting concentration of the SFN stock was 50 µM, and the starting concentration of the isothiocyanate standards was also 50 µM. SFN and its metabolites were quantified using an Agilent 6490 Triple Quadrupole mass spectrometer (Agilent Technologies, Santa Clara, CA, USA) with a HPLC Phenomenex Luna 3u C18(2) column (100-A, 100 × 2.1-mm) with a Phenomenex C18 100-A column guard. The system was comprised of a degasser, binary pump, column oven, cooled autosampler, diode array detector, and a 6490 MS. The LC-MS/MS was set at a flow rate of 0.3 mL/min. The column temperature and autosampler temperature were maintained at 40°C and 4°C, respectively. Samples were injected at 5 µL, system suitability was injected at 1 µL, and blanks were injected at 20 µL. Separation of metabolites was carried out with 0.1% ammonium acetate in Milli-Q water plus 0.1% acetic acid (mobile phase A, pH = 4) and 0.1% acetic acid in acetonitrile (mobile phase B). The LC eluent flow was sprayed into the mass spectrometer interface without splitting. Sulforaphane and conjugates were monitored using the MS Multiple Reaction Monitoring (MRM) mode in positive polarity with electrospray ionization source. The source parameters were gas temperature 200°C with a gas flow of 12 L/min, a sheath gas temperature of 400°C with a sheath gas flow of 12 L/min, a nebulizer pressure of 60 psi, and capillary voltage of 4000 V. Excel (Microsoft Corporation, Redmond, WA) was used for further data processing.

### GSH/GSSG-Glo Assay

FHL124 cells were seeded onto clear 96-well microtiter plates at a density of 5000 cells/well. Cell lysates were analyzed for glutathione using the Promega GSH/GSSG-Glo assay. Before the start of experimental conditions, cells were changed into EMEM without phenol red (Sigma-Aldrich) as instructed by the manufacturer to eliminate background signals. The total amounts of combined GSH and oxidized GSH (GSSG) were measured following the manufacturer's instruction. The luminescent signal was measured with the FLUOstar Omega microplate reader, and the GSH/GSSG ratio was calculated as [(net total glutathione RLU – net GSSG RLU)/(net GSSG RLU)] × 2, where RLU is relative light units. For human lens epithelium, match-paired halves of capsulorhexis samples were used. Two halves of capsulorhexis from the same eye would have the same treatment. This was because one half would be measured for the total GSH signal and the other half would be measured for the GSSG signal.

### Glutathione Reductase Activity

FHL124 cells were seeded onto 35-mm culture dishes until 95% confluency. The glutathione reductase (GR) activity was measured using the Glutathione Reductase Assay Kit (Abcam). At the end of the experiment, cells were washed once with cold Dulbecco's phosphate-buffered saline, and 300 µL of assay buffer was added. Cells were scraped using rubber policeman scrapers (Thermo Fisher Scientific), and the mix was collected into 1.5-mL Eppendorf tubes (Thermo Fisher Scientific). The mix was then homogenized on ice using a 2-mL Dounce homogenizer (Abcam) with 50 strokes for each sample. The lysate was transferred to a new 1.5-mL Eppendorf tubes and centrifuged at 13,000*g* for 15 minutes at 4°C. The supernatant was collected and stored at −80°C. The same protein amount (20 µg) quantified by the BCA assay was used for all samples. The GR activity was measured following the manufacturer's protocol with the FLUOstar Omega microplate reader.

### Statistical Analysis

Student's *t*-test analysis, paired or independent, was performed using Microsoft Excel to determine any statistical difference between two groups from the same donor or two different groups, respectively. One-way ANOVA with Tukey's post hoc analysis was used to assess multiple groups when all or many pairwise comparisons were of interest (SPSS Statistics 16.0; IBM, Chicago, IL, USA). One-way ANOVA with Dunnett's post hoc analysis (SPSS 16.0) was used to assess all groups compared against the control group. *P* ≤ 0.05 was considered significant.

## Results

### NAC Protects Against SFN-Induced Cell Death

**Figure 1.**
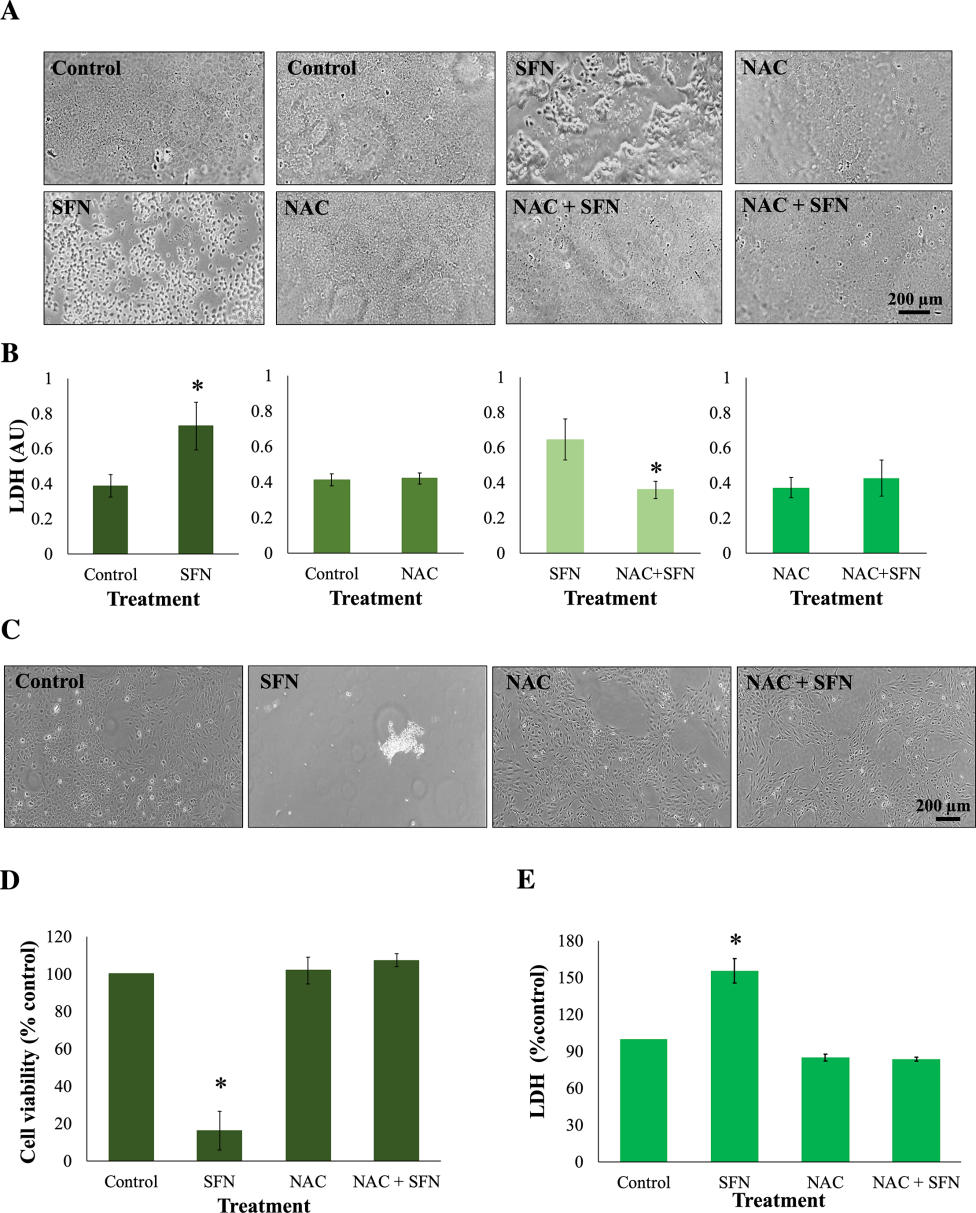
NAC protected human lens epithelium and FHL124 cells from SFN-induced cell death. Tissues or cells were maintained in either serum-free (control) EMEM or EMEM supplemented with 1-mM NAC. Following a period of 1 hour, half of the preparations were treated with SFN (final concentration 100 µM for tissues or 50 µM for cells) and the other half did not receive SFN treatment. The duration of SFN treatment was 18 hours. In human lens epithelium, (**A**) cell morphology was observed using a phase-contrast microscope, and (**B**) cytotoxicity was assessed by an LDH assay. In FHL124 cells, (**C**) cell morphology was observed using a phase-contrast microscope, (**D**) cell viability was measured using a Promega CellTiter-Glo assay, and (**E**) cytotoxicity was assessed by an LDH assay. Quantitative data were pooled from four match-paired human lens epithelium tissues or four separate experiments for FHL124 cells. Data are shown as mean ± SEM. Asterisks indicate a significant difference between the treated group and untreated controls (*P* ≤ 0.05, Student's paired *t*-test for human tissues, ANOVA with Tukey's post hoc test for FHL124 cells).

To investigate the role of reactive oxygen species (ROS) in SFN-induced cell death, NAC, a commonly used ROS scavenger, was employed to remove ROS from the cellular system. The concentration of 1-mM NAC was chosen from a range of concentrations offering cytoprotection (Supplementary Fig. S2.1), which is in accordance with several previous studies.^23^^,^^24^ Human lens epithelium or FHL124 cells were maintained in either serum-free (control) EMEM or EMEM supplemented with 1-mM NAC. Following a period of 1 hour, half of the preparations were treated with SFN (final concentration of 50 µM or 100 µM) and the other half did not receive SFN treatment. The presence of NAC did not reduce free SFN levels in the medium (Supplementary Fig. S2.2). Human lens epithelium treated with 100-µM SFN exhibited significant cell loss (Fig. 1A) and higher levels of lactate dehydrogenase (LDH) release (a marker of cellular damage) to the medium than untreated controls (Fig. 1B). Similar experiments were conducted in FHL124 cells, and NAC completely protected the cells from increased cell death (Fig. 1C), reduced cell viability (Fig. 1D), and increased cellular damage (Fig. 1E). NAC protected both the human lens epithelium and FHL124 cells from SFN-induced cell damage and cell death.

In addition, pyruvate, an end-product of glycolysis, has been used as a ROS scavenger in different cell lines,^25^^,^^26^ as increased concentrations of pyruvate can eliminate intracellular H_2_O_2_.^27^ In the current study, sodium pyruvate significantly protected FHL124 cells from cell loss induced by SFN (Supplementary Fig. S3.1). These findings suggest that ROS play an important role in SFN-induced cytotoxicity. As results between the human tissue and the cell line were comparable, FHL124 cells were used as the primary experimental model for the rest of the study in order to elucidate mechanistic events.

### SFN Causes Diverse Stress Responses Before Cell Death

**Figure 2.**
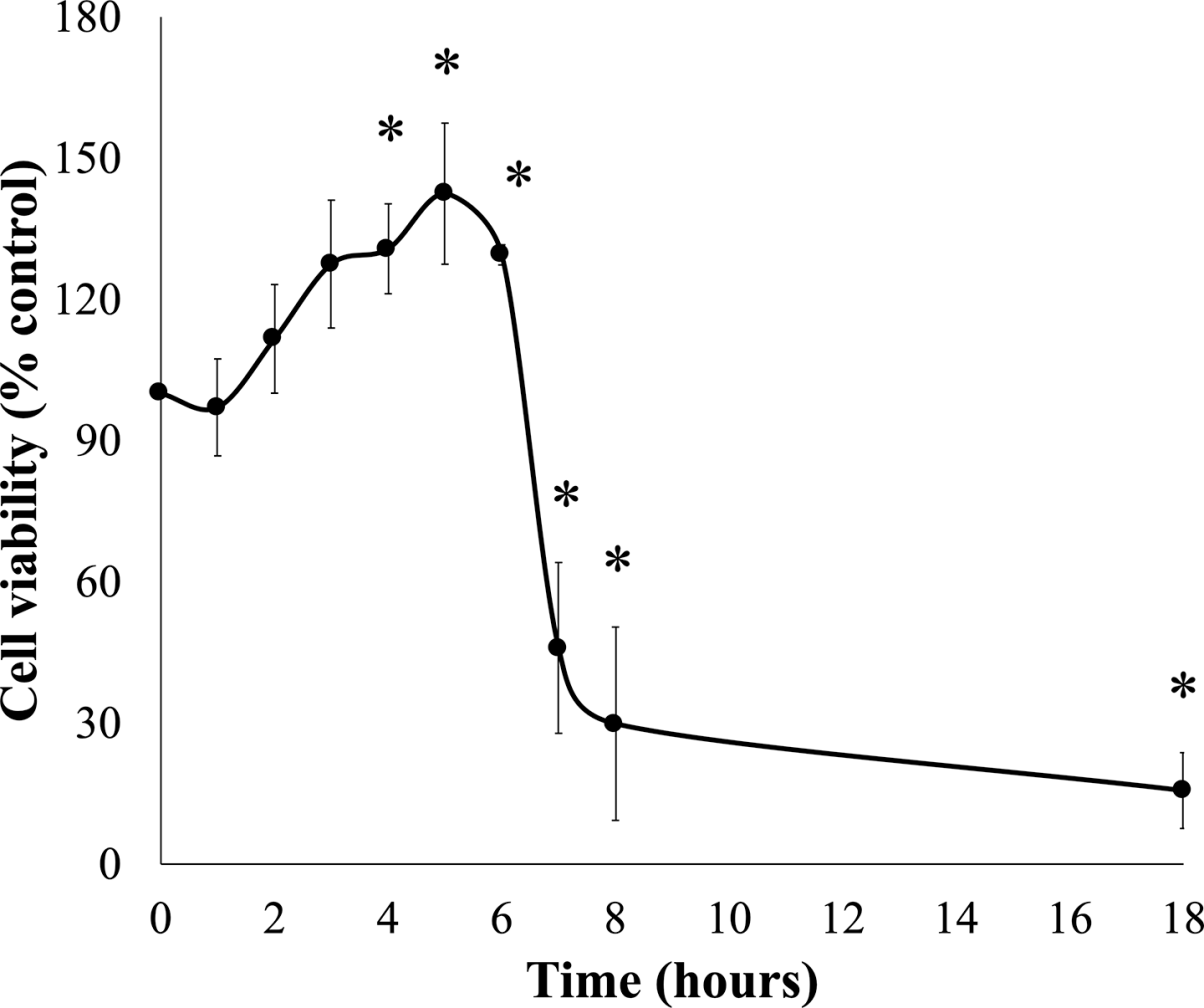
SFN decreased cell viability in FHL124 cells over time. FHL124 cells were treated with 50-µM SFN for different durations over the course of 18 hours and the cell viability was assessed using a Promega CellTiter-Glo assay. Quantitative data are shown as mean ± SEM (*n* = 4). Asterisks indicate a significant difference between the treated group and untreated controls at *t* = 0 (*P* ≤ 0.05, ANOVA with Dunnett's post hoc test).

**Figure 3.**
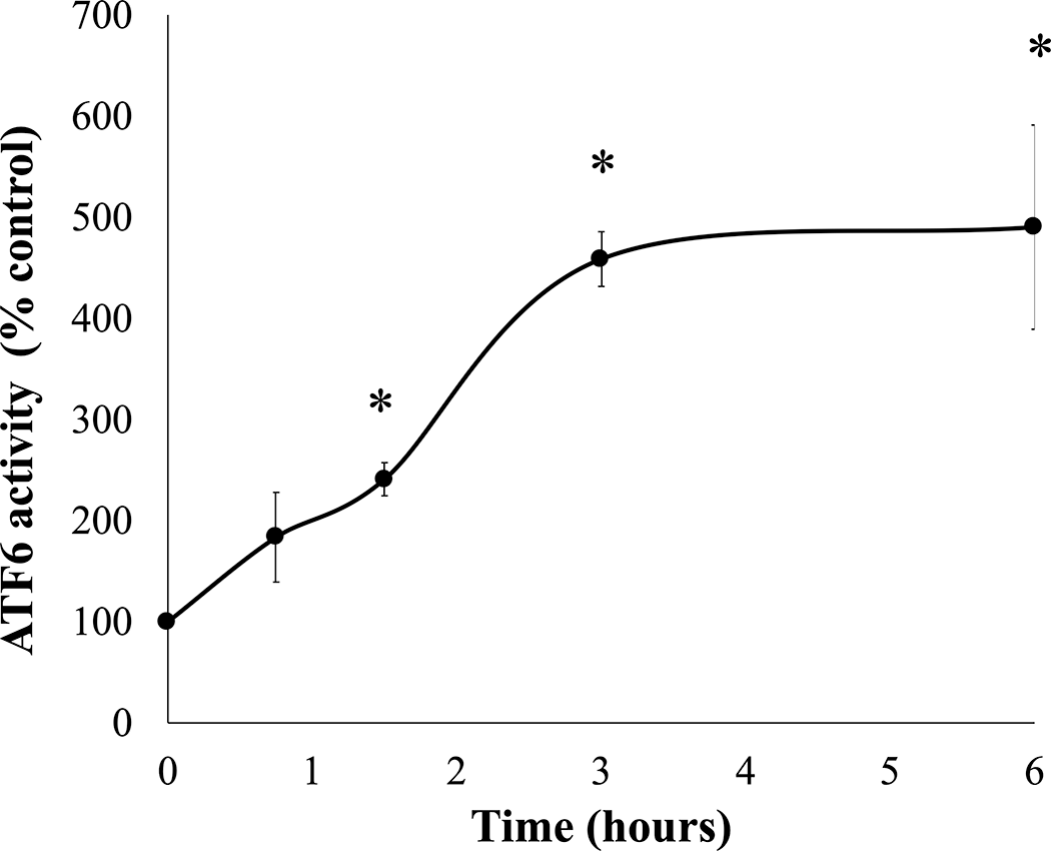
SFN increased ATF6 transcriptional activity in FHL124 cells over time. FHL124 cells were treated with 50-µM SFN for different durations over the course of 6 hours. The ATF6 transcriptional activity was measured using a Promega Dual-Luciferase Reporter Assay System. Quantitative data are shown as mean ± SEM (*n* = 4). Asterisks indicate a significant difference between the treated group and untreated controls at *t* = 0 (*P* ≤ 0.05, ANOVA with Dunnett's post hoc test).

**Figure 4.**
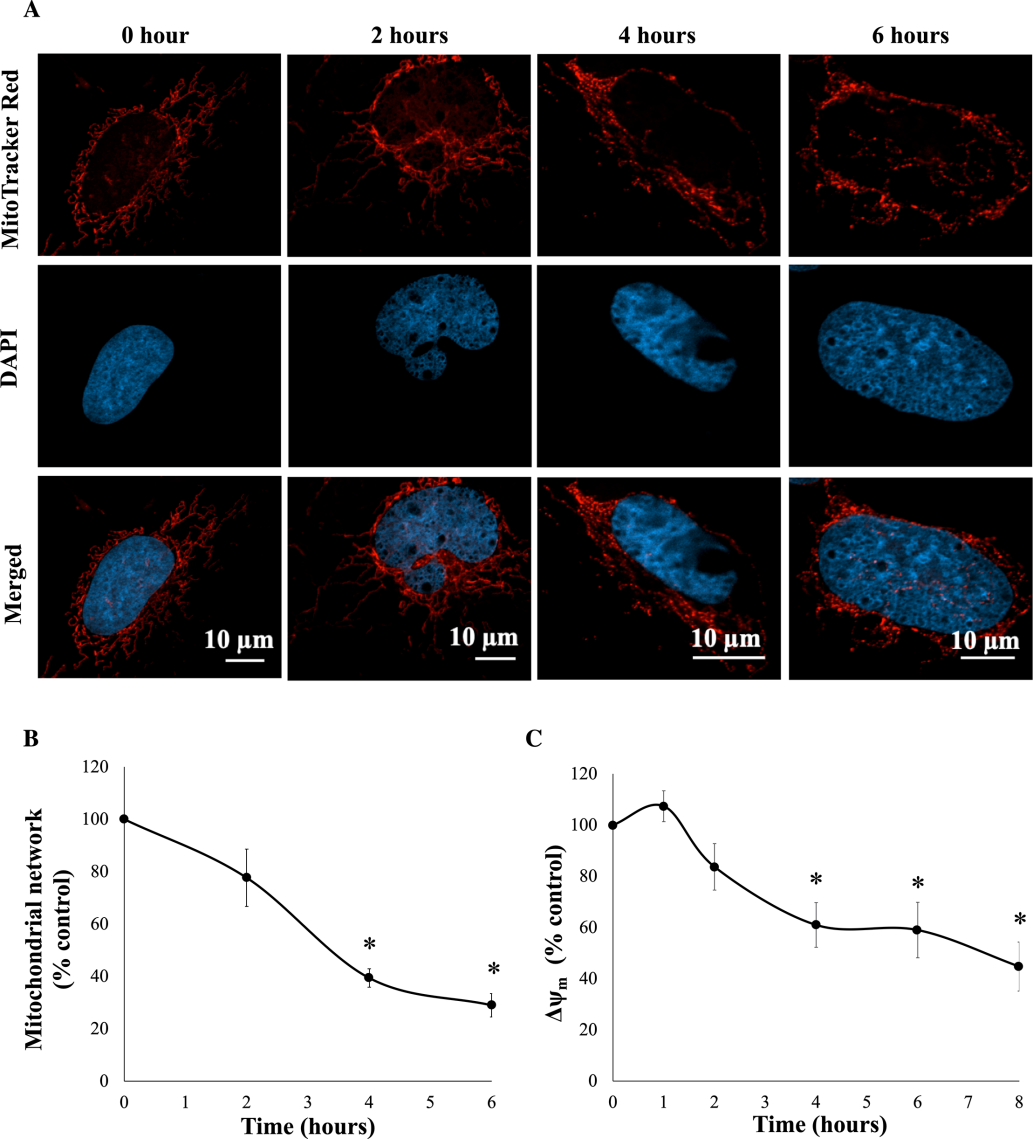
SFN increased mitochondrial damage in FHL124 cells over time. FHL124 cells were treated with 50-µM SFN for different durations over the course of 6 or 8 hours. (**A**) Representative images of mitochondria labeled with 100-nM MitoTracker Red CMXRos (*red*, MitoTracker Red; blue, DAPI; merged, MitoTracker Red and DAPI) were captured using a confocal microscope. (**B**) Quantitative data were pooled from four separate experiments and are shown as mean ± SEM (10 random cells/experiment). (**C**) For the mitochondrial membrane potential (Δψ_m_), FHL124 was incubated with 400-nM TMRE. The peak TMRE signal was measured using a microplate reader at Ex/Em = 549/575 nm. Quantitative data were pooled from four separate experiments and are shown as mean ± SEM. Asterisks indicate a significant difference between the treated group and untreated controls at *t* = 0 (*P* ≤ 0.05, ANOVA with Dunnett's post hoc test).

**Figure 5.**
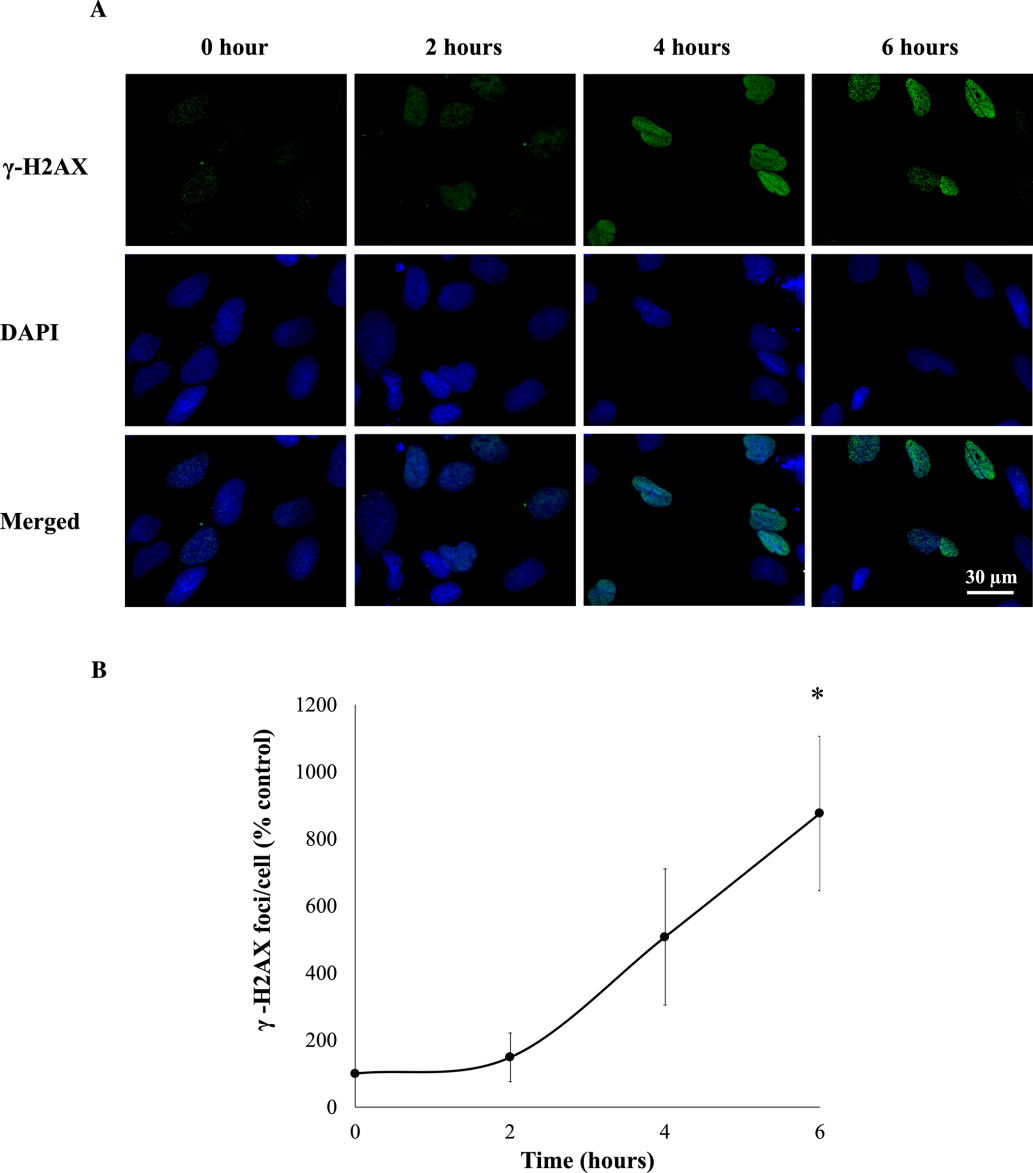
SFN increased DNA double-strand breaks in FHL124 cells over time. FHL124 cells were treated with 50-µM SFN for different durations over the course of 6 hours. γ-H2AX was used as a marker of DNA double-strand breaks using immunocytochemistry, such that foci per cell nucleus were determined by a widefield microscope. (**A**) Representative images (*green*, γ-H2AX; *blue*, DAPI; *merged*, γ-H2AX and DAPI) were captured with a widefield microscope, and (**B**) quantitative data are shown as mean ± SEM (*n* = 4). Asterisks indicate a significant difference between the treated group and untreated controls at *t* = 0 (*P* ≤ 0.05, ANOVA with Dunnett's post hoc test).

To further investigate cytotoxicity induced by 50-µM SFN, a series of cellular stress responses were assessed in time-response experiments. SFN caused and sustained diverse early stress responses that preceded cell death in FHL124 cells (Fig. 2). Previously, Liu et al.^11^ showed that SFN triggered ERS by upregulating levels of gene markers, such as *EIF2AK3*, *ATF6*, *HSPA5*, and *ERN1*, and of protein markers, such as BiP, ATF6, IRE1, and eiF2α.^11^ Here, the current study examined another marker of the ERS, the ATF6 transcriptional activity. After 6 hours, SFN induced a fivefold elevation (490.1% ± 101.0%) of ATF6 transcriptional activity against the baseline (Fig. 3). Mitochondrial dysfunctions were assessed by the size of mitochondrial networks and mitochondrial membrane potential (Δψ_m_). SFN significantly reduced the size of mitochondrial networks to 29.0% ± 4.5% of the control by 6 hours post-treatment (Figs. 4A, 4B) and decreased the Δψ_m_ by more than 50% (44.7% ± 9.5%) of the control by 8 hours post-treatment (Fig. 4C). DNA damage was measured by γ-H2AX foci as a marker of the DNA damage response to double-strand DNA breaks.^28^ At 6 hours post-treatment, the number of γ-H2AX foci was almost ninefold (875.6% ± 230.3%) higher than control values (Fig. 5).

### NAC Protects Against Diverse SFN-Induced Stress Responses

**Figure 6.**
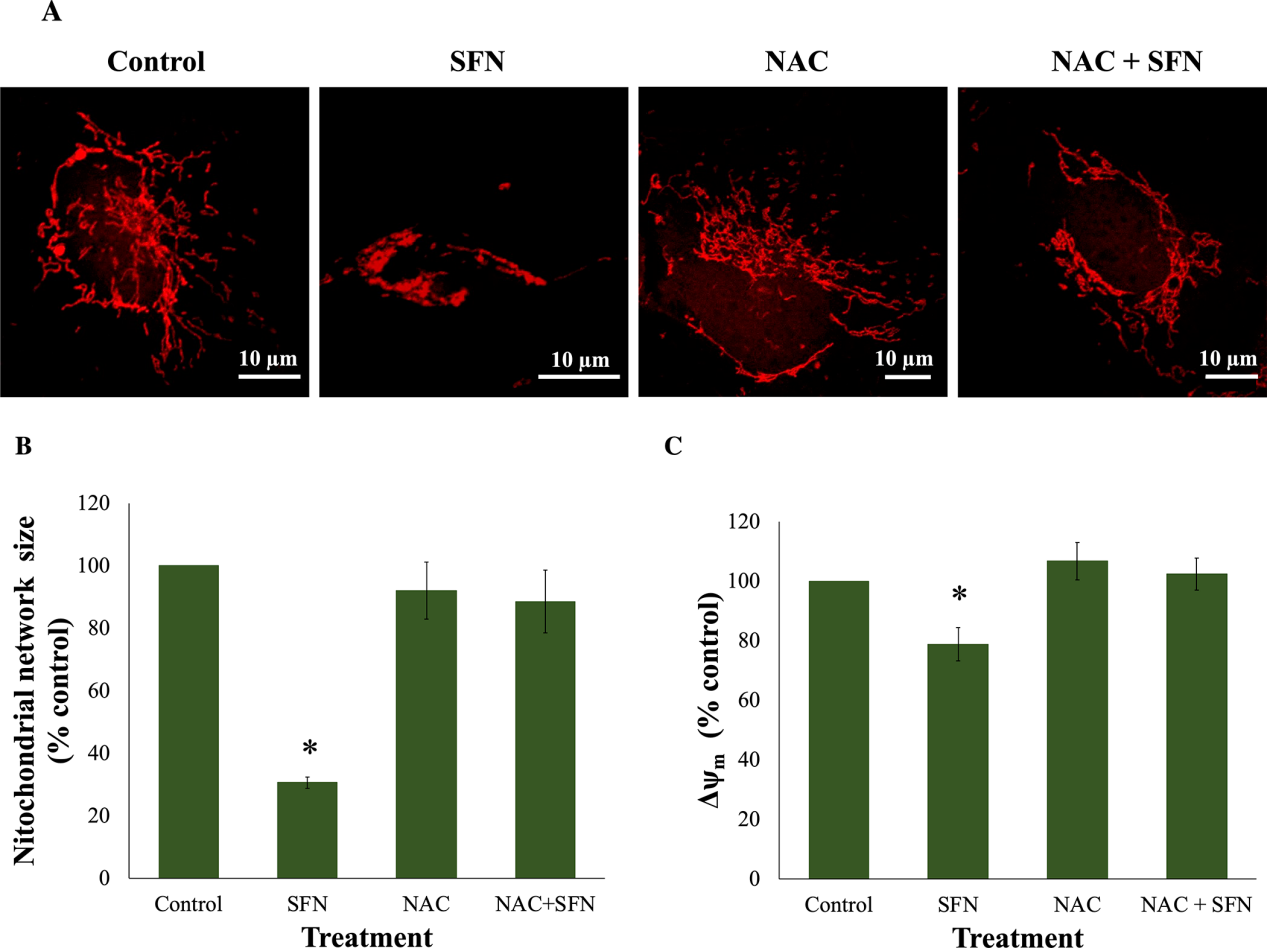
NAC protected cells from mitochondrial damage caused by SFN in FHL124 cells. Cells were maintained in either serum-free (control) EMEM or EMEM supplemented with 1-mM NAC. Following a period of 1 hour, half of the preparations were treated with SFN (final concentration 50 µM) and the other half did not receive SFN treatment. The duration of SFN treatment was 4 hours. (**A**) Representative images of mitochondria labeled with 100-nM MitoTracker Red CMXRos were captured with a confocal microscope, and (**B**) quantitative data pooled from three separate experiments are shown as mean ± SEM (20 random cells/experiment). (**C**) Mitochondrial membrane potential was measured as TMRE signal at Ex/Em = 549/575 nm, and the quantitative data were pooled from four separate experiments. Data are shown as mean ± SEM. Asterisks indicate a significant difference between the treated group and untreated controls (*P* ≤ 0.05; ANOVA with Tukey's post hoc test).

**Figure 7.**
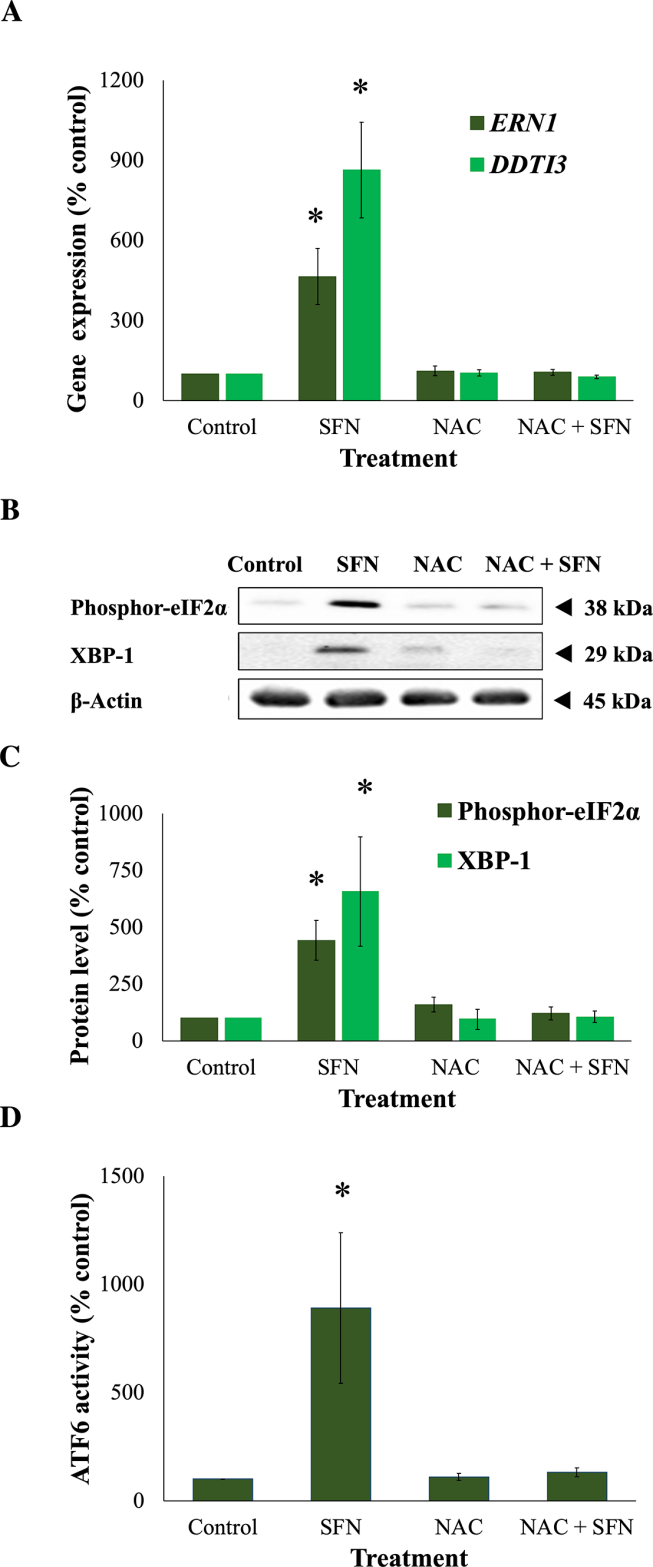
NAC prevented ERS responses propagated by SFN in FHL124 cells. Cells were maintained in either serum-free (control) EMEM or EMEM supplemented with 1-mM NAC. Following a period of 1 hour, half of the preparations were treated with SFN (final concentration 50 µM) and the other half did not receive SFN treatment. (**A**) Gene expression of two ERS gene markers (*ERN1*, encoding IRE1, and *DDTI3*, encoding CHOP) was measured after 18 hours of SFN treatment using TaqMan quantitative RT-PCR. The protein levels of two ERS protein markers (phosphor-EIF2⍺ and XBP-1) were examined after 18 hours of SFN treatment using western blot. (**B**) Representative gel from the western blot. (**C**) Quantification of the western blot data adjusted for β-actin loading controls. (**D**) The transcriptional activity of ATF6 was measured after 6 hours of SFN treatment by a Promega Dual-Luciferase Reporter Assay System. Quantitative data were pooled from three (**B**, **C**) or four (**A**, **D**) separate experiments and are shown as mean ± SEM. Asterisks indicate a significant difference between the treated group and untreated controls (*P* ≤ 0.05, ANOVA with Tukey's post hoc test).

**Figure 8.**
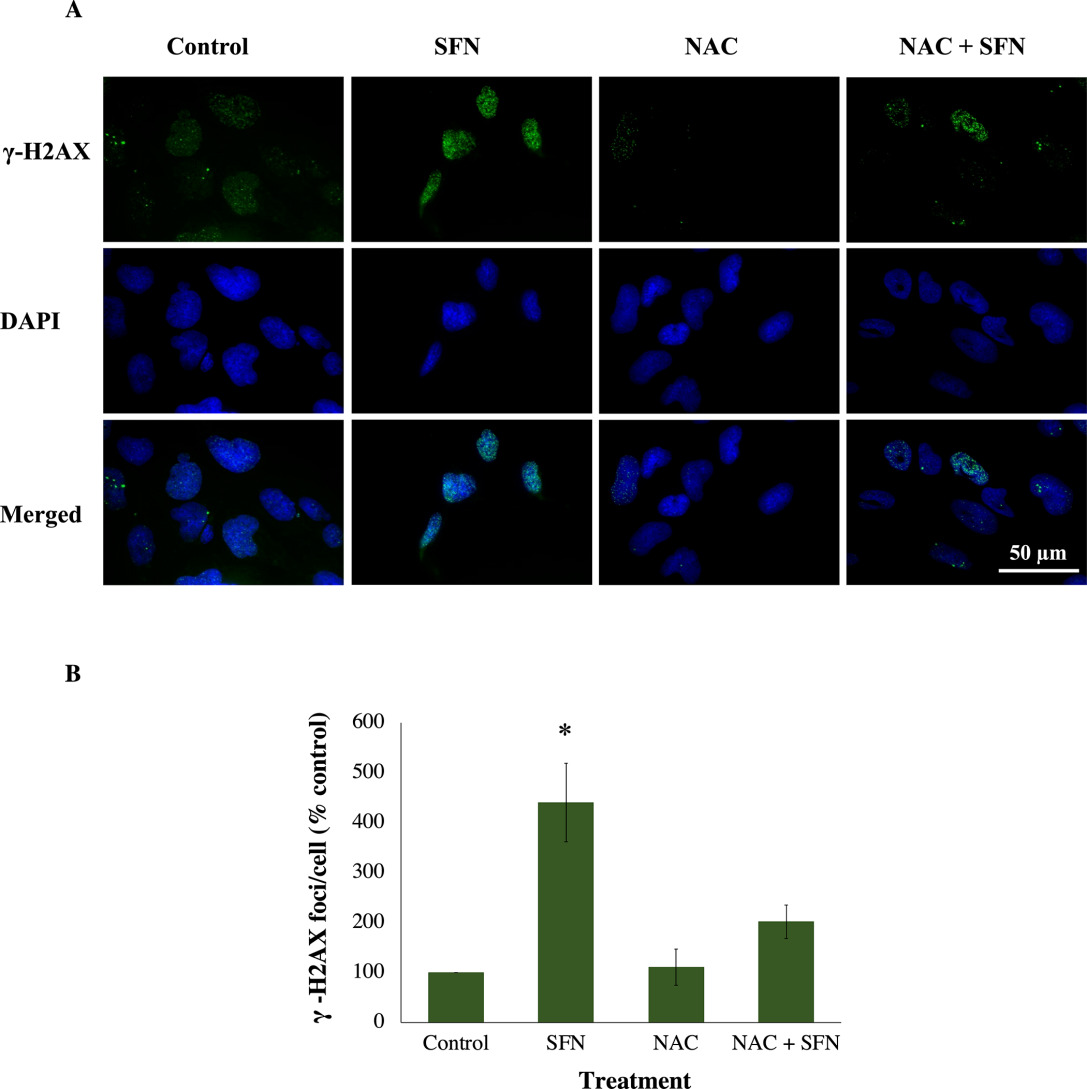
NAC prevented DNA double-strand breaks induced by SFN in FHL124 cells. Cells were maintained in either serum-free (control) EMEM or EMEM supplemented with 1-mM NAC. Following a period of 1 hour, half of the preparations were treated with SFN (final concentration 50 µM) and the other half did not receive SFN treatment. The duration of SFN treatment was 6 hours. (**A**) Representative images (*green*, γ-H2AX; *blue*, DAPI; *merged*, γ-H2AX and DAPI) were captured using a widefield microscope. (**B**) Quantitative data are shown as mean ± SEM (*n* = 4). Asterisks indicate a significant difference between the treated group and untreated controls (*P* ≤ 0.05, ANOVA with Tukey's post hoc test).

**Figure 9.**
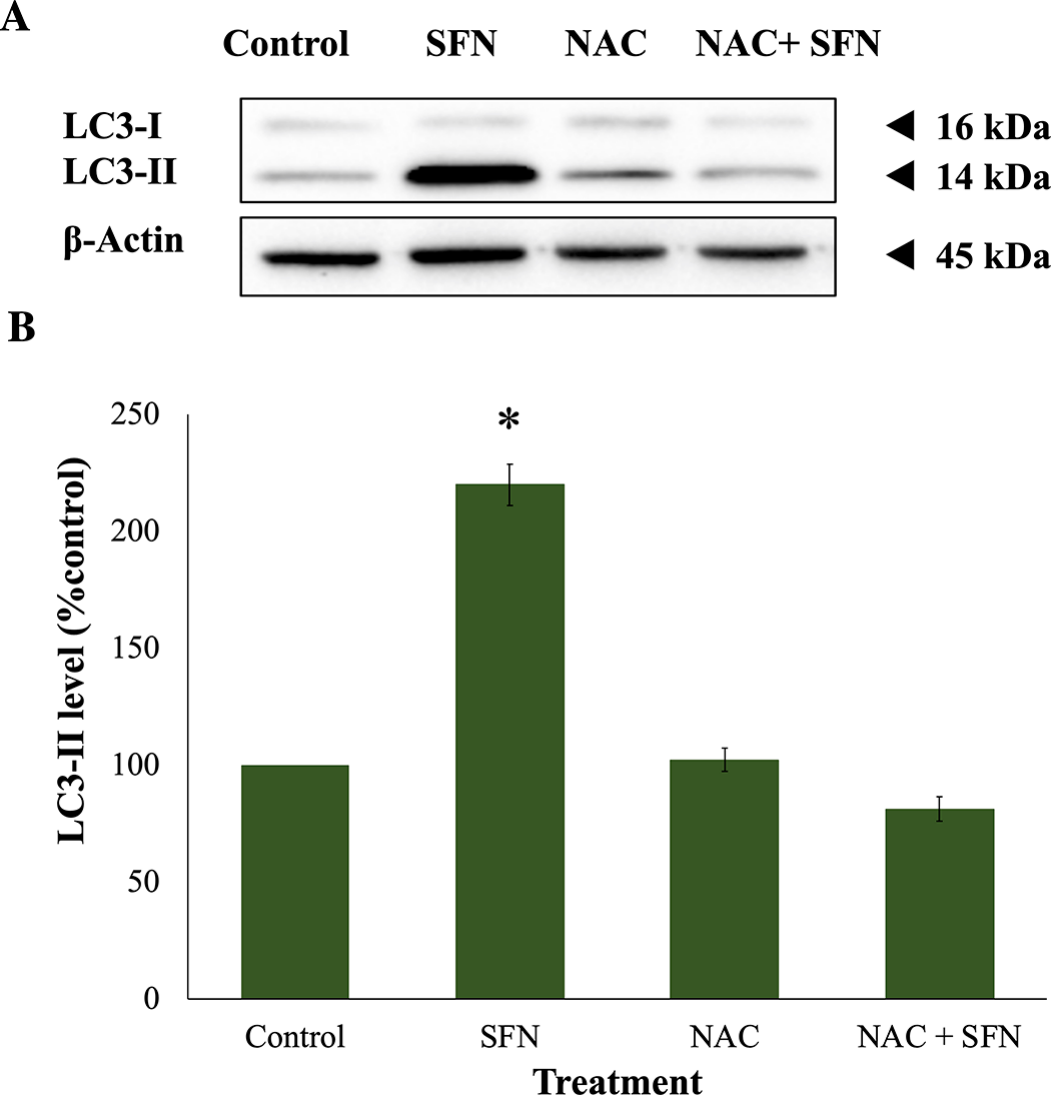
NAC protected autophagy induced by SFN in FHL124 cells. Cells were maintained in either serum-free (control) EMEM or EMEM supplemented with 1-mM NAC. Following a period of 1 hour, half of the preparations were treated with SFN (final concentration 50 µM) and the other half did not receive SFN treatment. Protein levels of autophagy marker LC3 after 18 hours of SFN treatment were measured by western blot. (**A**) Representative gel from the western blot. (B) Quantification of the western blot data adjusted for β-actin loading controls. Data are shown as mean ± SEM (*n* = 4). Asterisks indicate a significant difference between the treated group and untreated controls (*P* ≤ 0.05, ANOVA with Tukey's post hoc test for FHL124 cells).

Because SFN was shown to trigger various cellular stress pathways, it was important to investigate the potential protection of NAC against these responses. Specifically, NAC inhibited the collapse of mitochondrial networks (Figs. 6A, 6B) and the impairment of Δψ_m_ (Fig. 6C). Regarding ERS, NAC prevented the elevated expression of two ERS gene markers, *DDIT3* (encoding CHOP) and *ERN1* (encoding IRE1) (Fig. 7A); the increased levels of two ERS protein markers, phosphor-eiF2α and XBP1 (Figs. 7B, 7C); and the upregulated transcriptional activity of ATF6 (Fig. 7D). NAC helped maintain genomic integrity by limiting the elevation of γ-H2AX foci (Fig. 8). Autophagy is a major catabolic pathway that is kept at low basal levels and serves as the major intracellular degradation system for cell survival and renovation.^29^ However, autophagy also often accompanies cell death, usually upon extreme stress stimuli. ^30^ After 18 hours, 50-µM SFN significantly increased the protein levels of LC3-II, a marker of autophagy, compared to control cells without SFN treatment in FHL124 cells (Fig. 9). This is consistent with previous findings reported by Liu et al.^11^ NAC treatment prevented the elevation of autophagy. Loss of Δψ_m_ induced by SFN was also rescued by sodium pyruvate (Supplementary Fig. S3.2). This suggests that ROS play a key role in various SFN-induced stress responses in human lens cells.

### SFN Depletes the Intracellular GSH Pool in Human Lens Cells

**Figure 10.**
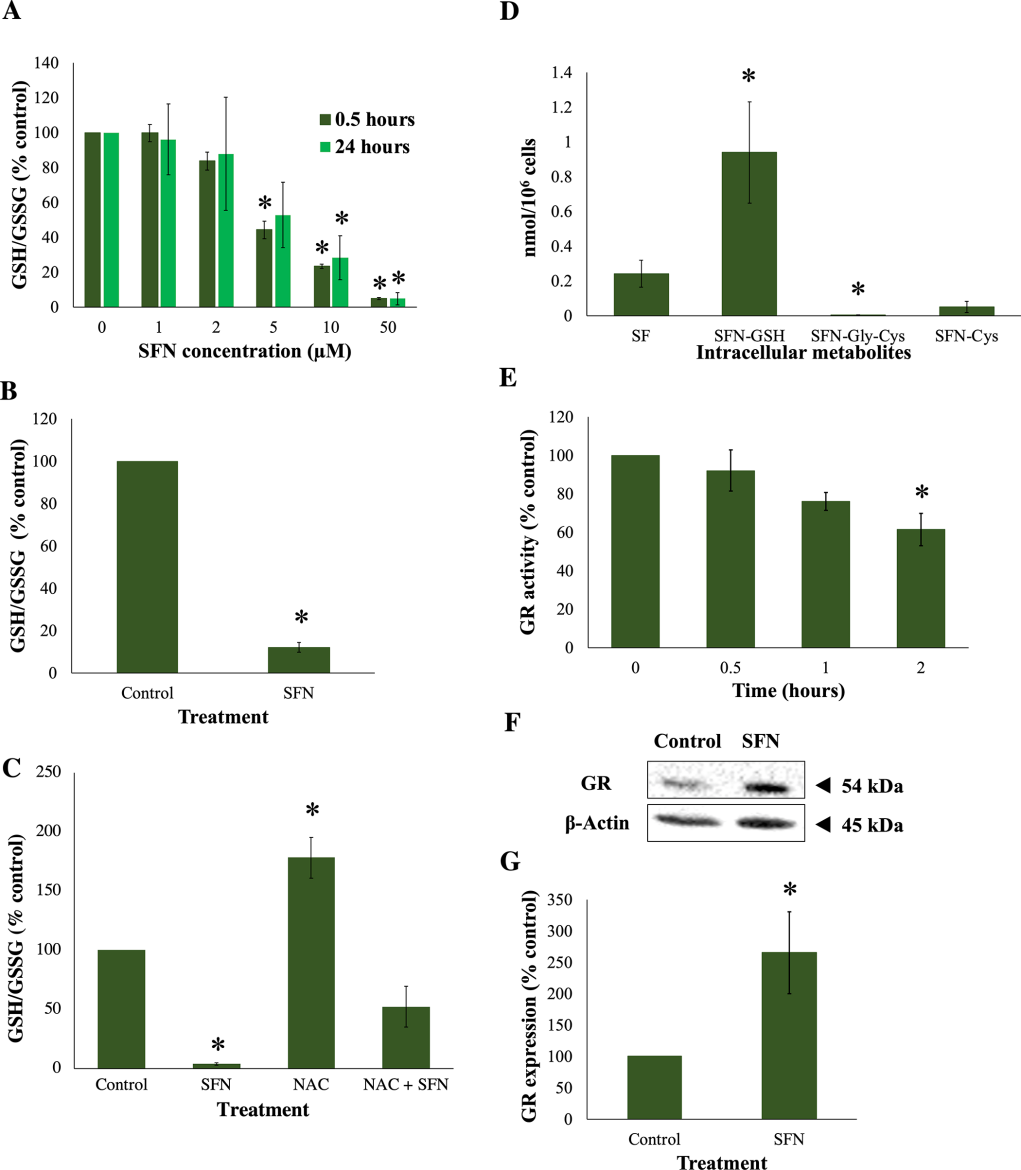
SFN impaired glutathione homeostasis. (**A**) FHL124 cells were treated with different concentrations of SFN for 0.5 and 24 hours, and GSH/GSSG was measured using a luminescent assay. (**B**) Human epithelium tissues from match-paired donors were treated with 50-µM SFN for 4 hours, and GSH/GSSG was measured similarly. (**C**) FHL124 cells were treated with 1-mM NAC before SFN as done before, and GSH/GSSG was measured after 24 hours. (**D**) Different intracellular metabolites of SFN in FHL124 cells after 1 hour of 50-µM SFN treatment were analyzed using LC-MS/MS. (E) Activity of GR of FHL124 cells treated with 50-µM SFN was measured using a GR activity kit. (F) Protein levels of GR in FHL124 cells after 18-hour 50-µM SFN treatment were measured by western blot. (**F**) Representative gel from western blot. (G) Quantification of the western blot data adjusted for β-actin loading controls. Quantitative data were pooled from three to four separate experiments for FHL124 cells and four match-paired human epithelium tissues. Data are shown as mean ± SEM. Asterisks indicate a significant difference between the treated group and untreated controls (*P* ≤ 0.05, ANOVA with Dunnett's post hoc test for **A**, **D**, and **E**, with the control groups being *t* = 0 for **A** and **E** or SFN metabolite for **D**; ANOVA with Tukey's post hoc test for **C**; Student's paired *t*-test for **B**; Student's independent *t*-test for **G**).

SFN has been reported to deplete GSH in several human experimental, largely cancer, models.^31^^–^^33^ To investigate the impacts of SFN on GSH in human lens cells, the GSH/GSSG ratio was measured. FHL124 cells were treated with different concentrations (0–50 µM) of SFN for different durations (0–24 hours) (Fig. 10A). The absolute ratio of GSH/GSSG in the control (*t* = 0, without SFN) was 25.3:1 (*n* = 3), which served as the baseline level. SFN treatment decreased the GSH/GSSG ratio in a concentration-dependent manner, and its dramatic effects were detected as early as after 30 minutes. At 24 hours post-treatment, slight increases in GSH/GSSG ratios were observed with most SFN concentrations; however, 50-µM SFN showed no increase and significantly maintained the ratio below 10% against the baseline level (4.8% ± 3.5%). Moreover, also at the endpoint, 50-µM SFN caused significant reductions in total glutathione and GSH levels (25.9 ± 1.9% and 5.7 ± 4.3%, respectively) and a significant increase in GSSG levels (168.7 ± 36.5%) relative to the control (Supplementary Fig. S4). In human lens epithelium, the absolute ratio of GSH/GSSG in the control was 13.9:1 (*n* = 3), which served as the baseline level. In this experimental model, 50-µM SFN also significantly depleted the GSH/GSSG ratio (12.1% ± 2.3%) (Fig. 10B). NAC almost doubled the GSH pool (178% ± 17%) but did not restore the GSH/GSSG level in FHL124 cells to the baseline level in the presence of SFN (52.3% ± 17.2%) (Fig. 10C).

### Mechanisms of SFN-Induced GSH Depletion

In light of SFN-induced depletion of GSH in lens cells, it was of interest to investigate what drives the phenomenon. One possible mechanism was the conjugation of GSH with SFN. Upon cellular entry, SFN reacts with GSH to form SFN–GSH conjugates (dithiocarbamates) under the catalytic activity of glutathione-*S*-transferase (GST). Subsequently, SFN–GSH will sequentially be converted to SFN–Gly–Cys, SFN–Cys, and SFN–NAC by γ-glutamyltransferase, cysteinylglycinase, and *N*-acetyltransferase, respectively, in the mercapturic acid pathway.^4^^,^^34^ To investigate this, LC-MS/MS analysis was used to measure different intracellular metabolites of SFN in FHL124 cells. After 1 hour of SFN addition, SFN–GSH was shown to be the most prominent intracellular metabolite (no detection of SFN–NAC) (Fig. 10D). Glutathione reductase is the enzyme catalyzing the conversion of GSSG back to GSH, and its activity helps maintain the intrinsic high GSH/GSSG level. Under 50-µM SFN, GR activity was reduced to 66.9% ± 5.6% after 2 hours (Fig. 10E) and continued to decrease further, such that after 18 hours the activity was 40.6% ± 10.1% of the untreated control (Supplementary Fig. S5). In contrast, protein levels of GR were increased by more than twofold (265.4% ± 65.3%) at 18 hours post-SFN (Figs. 10F, 10G). The data suggest that SFN depleted GSH by conjugating with GSH and decreasing GR activity.

### GSH Supplementation Protects Against SFN-Induced Cytotoxicity

**Figure 11.**
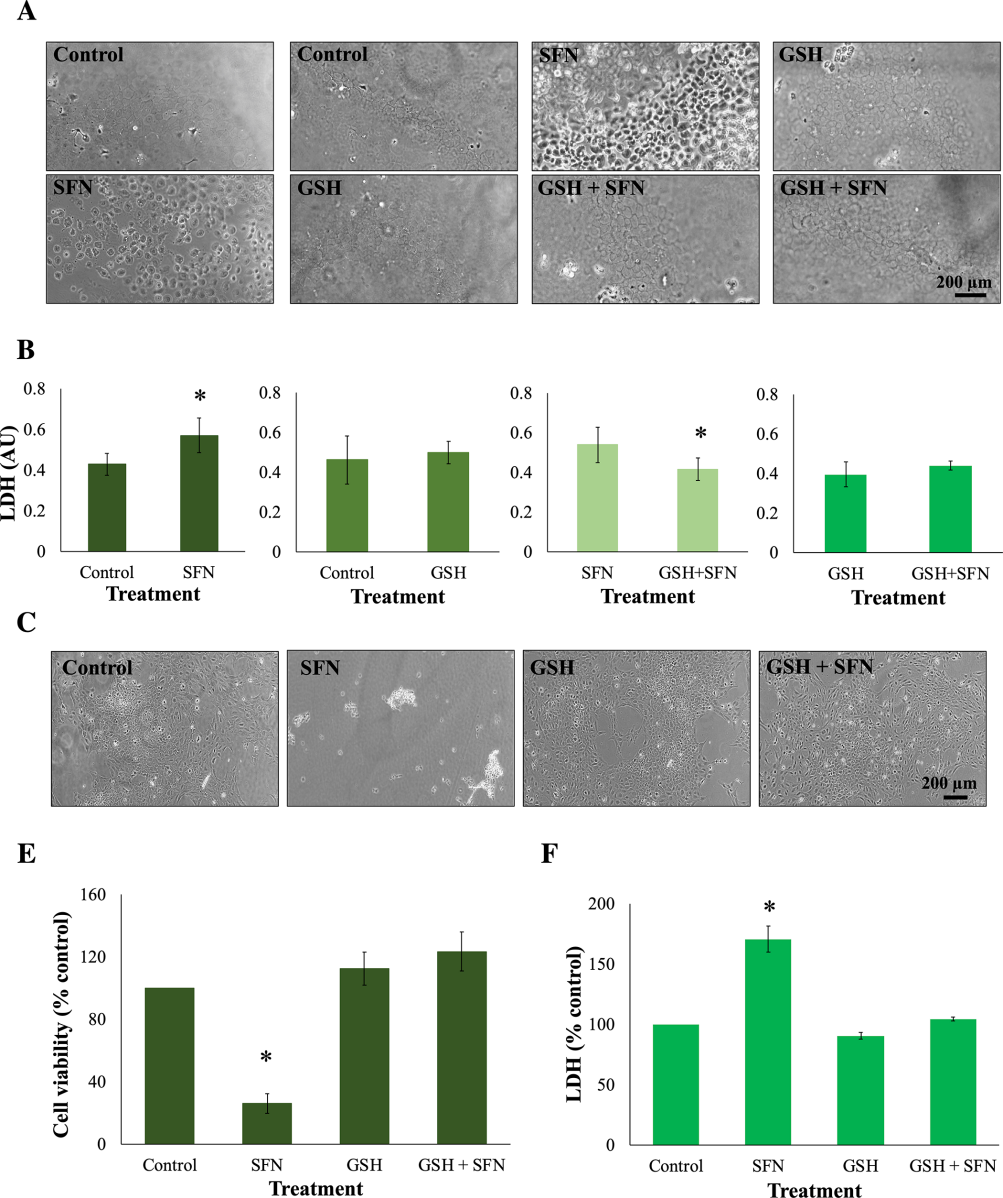
GSH protected human lens epithelium and FHL124 cells from SFN-induced cell death. Tissues or cells were maintained in either serum-free (control) EMEM or EMEM supplemented with 1-mM GSH. Following a period of 1 hour, half of the preparations were treated with SFN (final concentration 100 µM for tissues or 50 µM for cells) and the other half did not receive SFN treatment. The duration of SFN treatment was 18 hours. In human lens epithelium, (**A**) cell morphology was observed using a phase-contrast microscope, and (**B**) cytotoxicity was assessed using an LDH assay. In FHL124 cells, (**C**) cell morphology was observed using phase-contrast microscopy, (**D**) cell viability was measured using a Promega CellTiter-Glo assay, and (**E**) cytotoxicity was assessed by an LDH assay. Quantitative data were pooled from four match-paired human lens epithelium tissues or four separate experiments for FHL124 cells. Data are shown as mean ± SEM. Asterisks indicate a significant difference between the treated group and untreated controls (*P* ≤ 0.05, Student's paired *t*-test for human tissues, ANOVA with Tukey's post hoc test for FHL124 cells).

**Figure 12.**
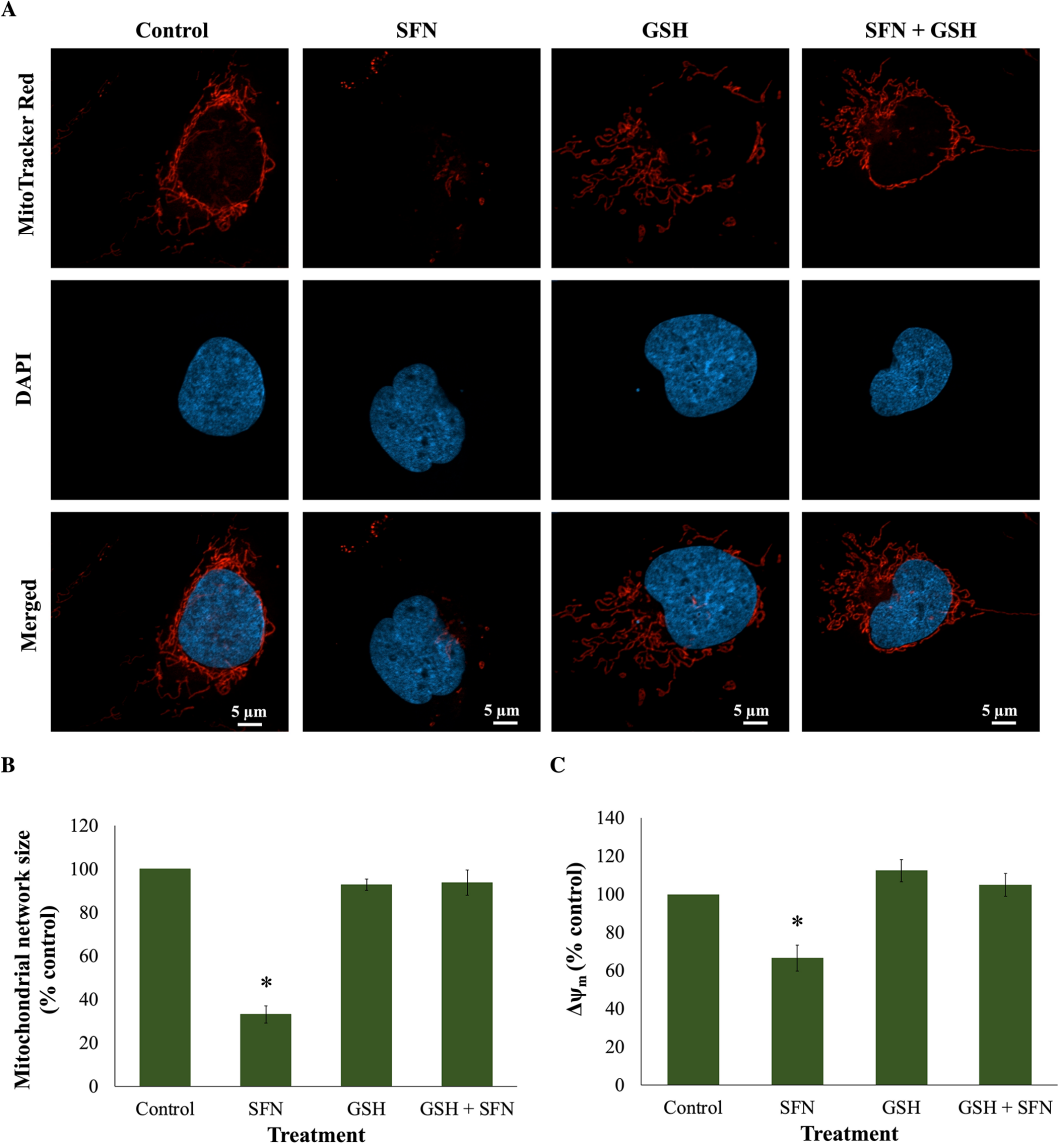
GSH protected cells from mitochondrial network collapse caused by SFN in FHL124 cells. Cells were maintained in either serum-free (control) EMEM or EMEM supplemented with 1-mM GSH. Following a period of 1 hour, half of the preparations were treated with SFN (final concentration 50 µM) and the other half did not receive SFN treatment. The SFN treatment lasted for 4 hours. (**A**) Representative images of mitochondria labeled with 100-nM MitoTracker Red CMXRos were captured using a confocal microscope. (**B**) Quantitative data were pooled from four separate experiments and are shown as mean ± SEM (10 random cells/experiment). (**C**) Mitochondrial membrane potential was measured as TMRE signal at Ex/Em = 549/575 nm, and quantitative data were pooled from four separate experiments. Data are shown as mean ± SEM. Asterisks indicate a significant difference between the treated group and untreated controls (*P* ≤ 0.05, ANOVA with Tukey's post hoc test).

**Figure 13.**
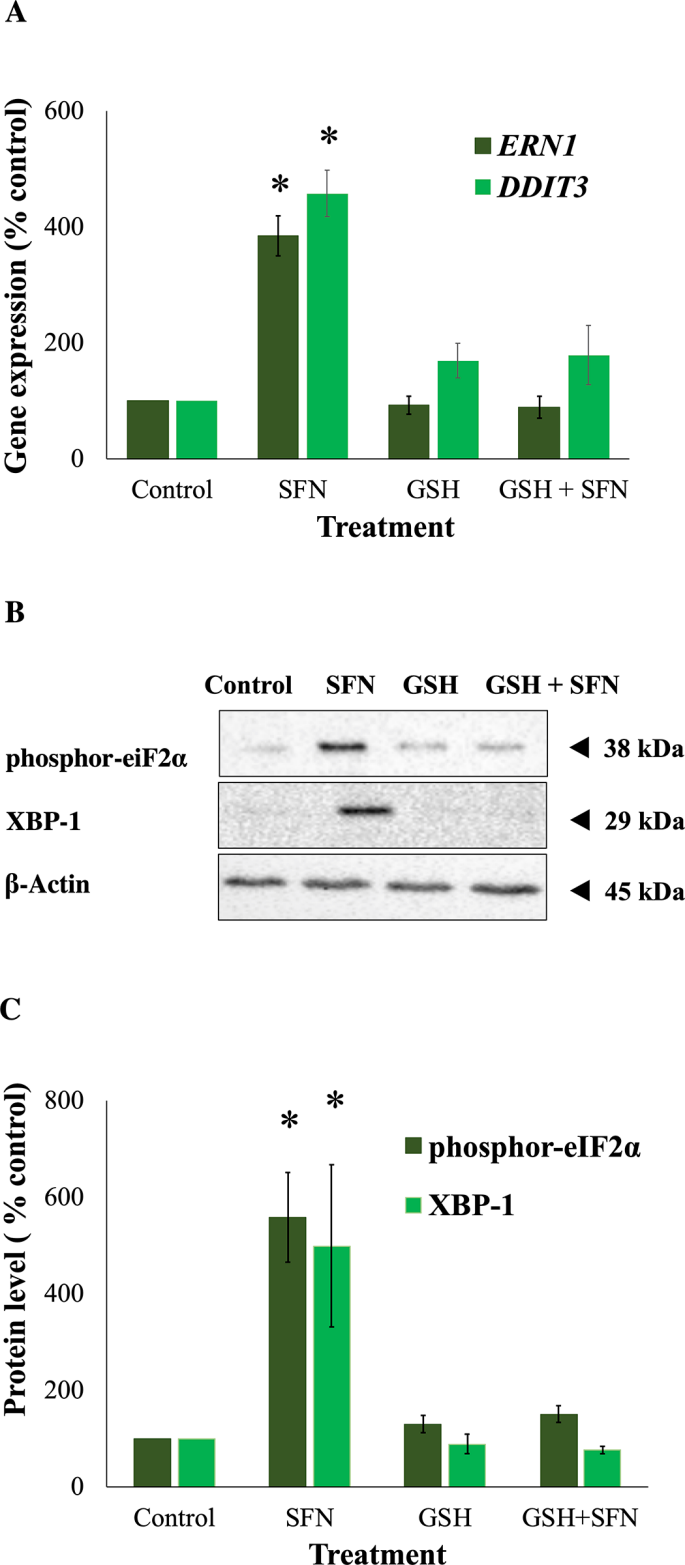
GSH prevented ERS responses propagated by SFN in FHL124 cells. Cells were maintained in either serum-free (control) EMEM or EMEM supplemented with 1-mM GSH. Following a period of 1 hour, half of the preparations were treated with SFN (final concentration 50 µM) and the other half did not receive SFN treatment. (**A**) Gene expression of two ERS gene markers (*ERN1*, encoding IRE1, and *DDTI3*, encoding CHOP) was measured after 18 hours of SFN treatment using TaqMan quantitative RT-PCR. Protein levels of two ERS protein markers (phosphor-EIF2⍺ and XBP-1) were examined after 18 hours of SFN treatment using western blot. (**B**) Representative gel from the western blot. (**C**) Quantification of the western blot data adjusted for β-actin loading controls. Quantitative data were pooled from four (**A**) or three (**B**, **C**) separate experiments and are shown as mean ± SEM. Asterisks indicate a significant difference between the treated group and untreated controls (*P* ≤ 0.05, ANOVA with Tukey's post hoc test).

**Figure 14.**
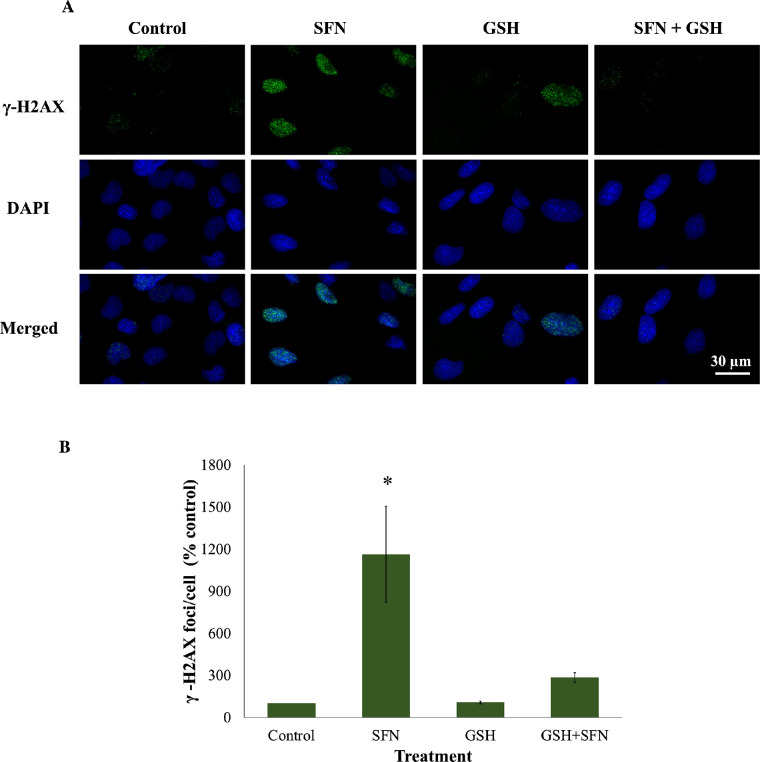
GSH prevented DNA double-strand breaks induced by SFN in FHL124 cells. Cells were maintained in either serum-free (control) EMEM or EMEM supplemented with 1-mM GSH. Following a period of 1 hour, half of the preparations were treated with SFN (final concentration 50 µM) and the other half did not receive SFN treatment. The duration of SFN treatment was 6 hours. (**A**) Representative images (*green*, γ-H2AX; *blue*, DAPI; *merged*, γ-H2AX and DAPI) were captured using a widefield microscope. (**B**) Quantitative data are shown as mean ± SEM (*n* = 4). Asterisks indicate a significant difference between the treated group and untreated controls (*P* ≤ 0.05, ANOVA with Tukey's post hoc test).

**Figure 15.**
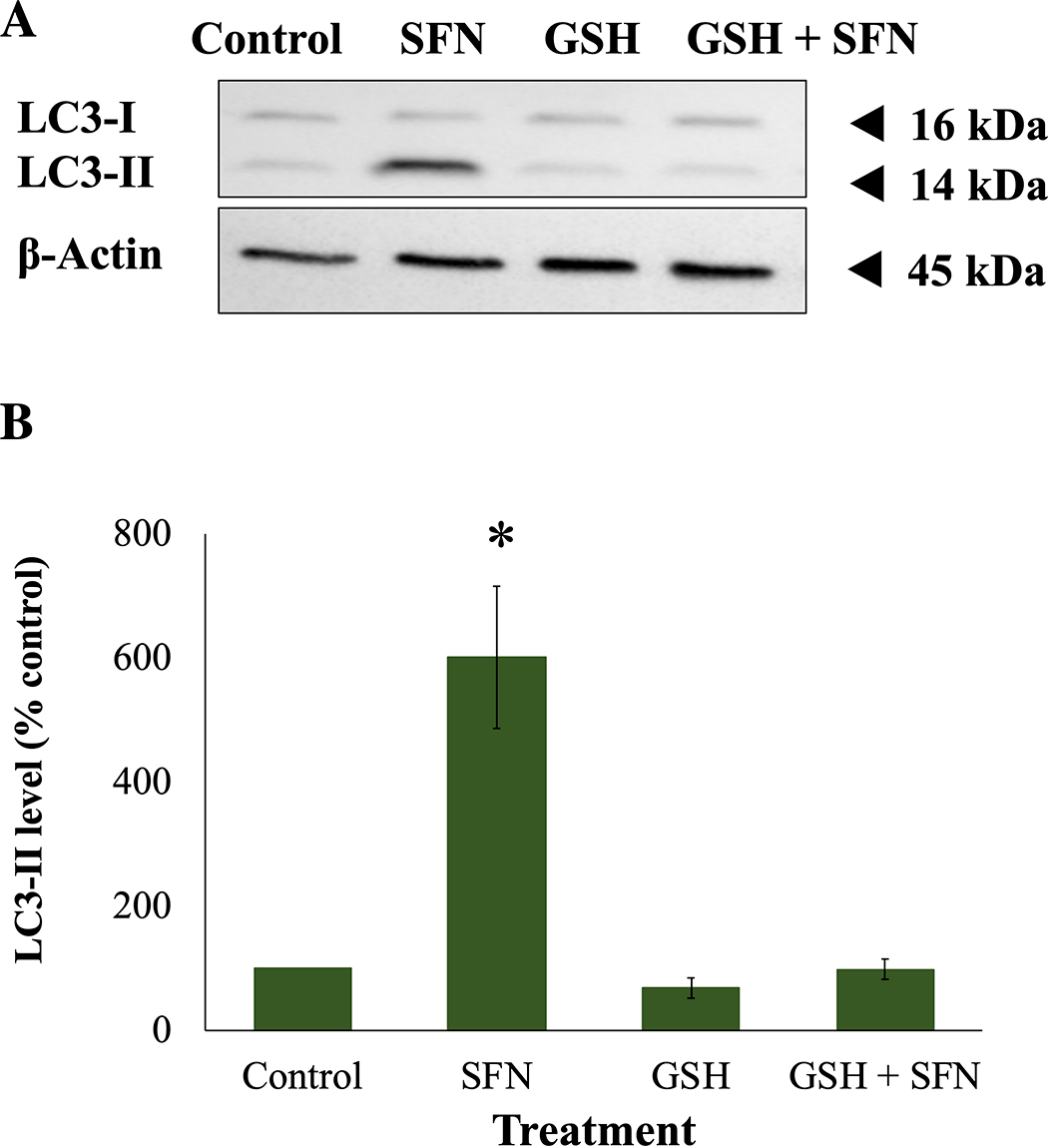
GSH protected against autophagy induced by SFN in FHL124 cells. Cells were maintained in either serum-free (control) EMEM or EMEM supplemented with 1-mM GSH. Following a period of 1 hour, half of the preparations were treated with SFN (final concentration 50 µM) and the other half did not receive SFN treatment. Protein levels of autophagy marker LC3 after 18 hours of SFN treatment were measured using western blot. (**A**) Representative gel from the western blot, and (**B**) quantification of the western blot data adjusted for β-actin loading controls. Data are shown as mean ± SEM (*n* = 4). Asterisks indicate a significant difference between the treated group and untreated controls (*p* ≤ 0.05, ANOVA with Tukey's post hoc test for FHL124 cells).

GSH depletion was shown to be one of the early effects of SFN in human lens cells, and this led to a question of which role this event plays in cytotoxicity induced by SFN. Similar to the previous investigation of the role of ROS, GSH was used instead of NAC or sodium pyruvate. The GSH supplementation did not decrease the free SFN levels present in the culture medium (Supplementary Fig. S2.2) and, importantly, significantly increased the intracellular GSH levels by threefold compared with the baseline level after 2 hours (Supplementary Fig. S6). The addition of 1-mM GSH protected human lens epithelium and FHL124 cells from cellular damage and cell death induced by SFN (Fig. 11). GSH further protected FHL124 cells from SFN-induced mitochondrial network collapse and loss of Δψ_m_ (Fig. 12), ERS (Fig. 13), DNA damage (Fig. 14), and autophagy (Fig. 15). Collectively, such findings indicate that GSH depletion plays a pivotal role in SFN-induced cytotoxicity.

## Discussion

SFN is a molecule of growing importance in medicine. It has been proposed as an agent to treat a number of conditions ranging from cancer to metabolic conditions.^7^^–^^10^ However, the mechanisms governing SFN-induced cell death are poorly understood in non-cancer cells. In the present study, we utilized the powerful experimental attributes of human lens cells and tissue^35^ to elucidate a chain of events that links SFN exposure to ultimate cell death.

Previously, it has been proposed that, because of its cytotoxicity, SFN could serve as a potential treatment to prevent the common lens disorder PCO, which affects a large number of cataract patients.^11^ The present study shows that oxidative stress arising from SFN-induced GSH depletion and ROS formation triggers various stress responses and eventually cell death in human lens cells.

We demonstrated that treatment with two ROS scavengers, NAC and sodium pyruvate, protected cells from death, suggesting that increased ROS levels play a key role in SFN-induced death in FHL124 cells. In several cancer cell lines, SFN-induced cell cycle arrest or cell death was also prevented by alleviating ROS levels, such as by overexpressing catalase, an endogenous antioxidant, or supplementing cells with NAC or (2R, 4R)-4-aminopyrrolidine-2,4-dicarboxylate (ADPC), a ROS inhibitor.^23^^,^^36^^–^^39^ The Nrf2 pathway regulates detoxification and antioxidant defense processes, and SFN is a potent activator of Nrf2.^4^ Nonetheless, the elevation of Nrf2 activity beyond physiological levels can be detrimental to cells. In another human lens cell line, high levels of SFN-induced ROS were reported to cause Nrf-2 dependent activation of Kruppel-like factor 9, which further fueled the ROS production and promoted oxidative stress-induced cell death.^40^

The complete protection of NAC from other SFN-induced cellular events, such as mitochondrial dysfunction, ERS, DNA damage, and autophagy, and the protection yielded by sodium pyruvate against impaired Δψ_m_ collectively indicate an important role for ROS in these SFN-induced phenomena. The current findings are comparable to those of previous studies, in which SFN induced ROS-dependent Δψ_m_ perturbations in human leukemia cells,^41^ ROS-dependent DNA damage and responses in umbilical vein endothelial cells and esophageal cancer cells,^39^^,^^42^ and ROS-dependent autophagy in colon, prostate, and esophageal cancer cells.^37^^,^^39^^,^^43^ Moreover, although others have demonstrated the induction of ERS by SFN,^11^^,^^44^ the current work provides the first evidence that the induction is ROS dependent. These cellular events individually may not suffice to induce cell death, and some can also be anti-apoptotic.^45^^–^^47^ Nonetheless, given their chronicity and intensity, it is likely that they collectively contribute to SFN-induced cell death. Therefore, our data provide compelling evidence that, via increasing ROS levels, SFN induces various cellular stress responses that lead to cellular demise.

Intracellular ROS levels are tightly regulated by a multilayered antioxidant defense system to maintain redox homeostasis. Following SFN exposure, human lens cells experienced increased ROS levels and subsequently a cascade of ROS-dependent detrimental phenomena. This prompted an investigation into the possible effects of SFN on the antioxidant defense. GSH is the master antioxidant in lens epithelial cells, where it can have exceptionally high concentrations of 4 to 6 mM,^48^ compared with 1 to 2 mM in most cells.^49^ Its ratio with GSSG reflects a cellular redox status. In a resting cell, the GSH/GSSG ratio is relatively high because GSH is the dominant form of intracellular glutathione. However, following oxidative stress, this ratio can be significantly reduced, even to 1:1.^50^^–^^52^ We showed that after a 30-minute exposure to 50-µM SFN, the GSH/GSSG ratio in FHL124 cells was decreased to below 10% of the baseline level, which was typically in the region of 25:1, and did not recover after 24 hours. This remarkably low GSH/GSSG ratio indicates chronic oxidative stress in human lens cells upon the administration of 50-µM SFN.

Moreover, there was a clear distinction in GSH/GSSG ratios between non-cytotoxic concentrations and cytotoxic concentrations. Low concentrations of SFN (1 and 2 µM), previously shown to be non-cytotoxic,^5^ maintained the decreased GSH/GSSG ratios above 80%, whereas the high, supranutritional concentration (50 µM), previously shown to be cytotoxic, decreased the ratios to less than 10% of the baseline. This indicates a possible correlation between SFN-induced GSH depletion and cytotoxicity. GSH depletion is also an early apoptosis hallmark in varying human cell lines.^53^^–^^55^ Therefore, in our human lens model, the rapid depletion of GSH by SFN appears to trigger SFN-induced cell death.

We discovered two mechanisms that cause GSH depletion. One mechanism is the GST-catalyzed conjugation between SFN and GSH in the mercapturic acid pathway, which was evidenced by the LC-MS/MS analysis showing SFN–GSH as the predominant intracellular SFN metabolite. This finding aligns with the current literature, which has reported that, upon entry into mammalian cells, SFN can form a thioether bond with GSH in a reaction catalyzed by GST as the initial step of its metabolism via the mercapturic acid pathway.^56^^–^^58^ Moreover, the major depletion of degradation-resistant GSH is through conjugation with endogenous and xenobiotic compounds, such as SFN. ^34^^,^^59^^,^^60^ Interestingly, no SFN–NAC was detected using LC-MS/MS. SFN–NAC is converted from SFN–Cys by arylamine *N*-acetyltransferase (NAT) in the final step of the metabolic mercapturic acid pathway.^4^ The metabolite panel was analyzed after 1 hour of SFN addition, which could be too early for the formation of SFN–NAC or for SFN–NAC levels to reach the detectable threshold. Moreover, human NAT has been reported to be rapidly inactivated by physiological concentrations of H_2_O_2_, and GSH can reverse this inactivation.^61^ With respect to the rapid depletion of GSH after 30 minutes, NAT was possibly disabled, resulting in the inefficient conversion of SFN–Cys to SFN–NAC.

The other mechanism of GSH depletion is reduced regeneration by decreased GR activity; despite doubled protein levels, GR activity dropped by nearly 40%. The decline in GR activity was likely due to both direct and indirect mechanisms. SFN can directly interact with GR, which leads to irreversible modifications and reduced GR activity.^62^ Additionally, GR regenerates GSH from GSSG at the expense of NAD(P)H. In FHL124 cells, SFN has been shown to induce NAD(P)H quinone oxidoreductase 1 (NQO1),^5^ which also oxidizes NAD(P)H to NAD(P)^+^, hence decreasing the NAD(P)H levels. Therefore, indirectly, SFN can debilitate GR activity by depleting the NAD(P)H pool. The increased quantity of GR can be interpreted as a compensatory effort.

The GR-dependent GSH regeneration is important to maintaining high GSH levels. In particular, GR function is critical to maintaining GSH levels within organelles that lack the synthesis machinery, such as mitochondria and the ER.^63^ Thus, the activity deficiency might directly exacerbate the already low GSH levels. This localized depletion of GSH in effect renders these organelles vulnerable to oxidative stress, leading to dysfunction. We, therefore, propose that GR inhibition is a major contributing factor to GSH depletion within SFN-treated cells. Previous work has shown that application of the GSH synthesis inhibitor buthionine sulfoximine does not lead to lens cell death but does sensitize cells to stress.^64^^,^^65^ This interesting disparity in outcome may lie at the organelle level. Therefore, future studies should aim to elucidate the impact of GSH-disrupting agents on the entire cell and specific organelles.

The key role of GSH depletion in SFN-induced stress responses and cytotoxicity was demonstrated by the complete protection yielded by GSH supplementation. SFN is a reactive electrophile, and both GSH and NAC are reactive nucleophiles. Therefore, GSH and NAC have the potential to spontaneously form conjugates with SFN in the medium, thus preventing the uptake of SFN. However, non-enzymatic reactions are 10^3^- to 10^5^-fold less efficient than GST-catalyzed reactions that take place inside the cell.^34^ Therefore, extracellular, non-enzymatic conjugation of SFN with NAC or GSH should be negligible and should not interfere with the cellular uptake of SFN. Additionally, using mass spectrometry, we confirmed that both NAC and GSH did not decrease the levels of free SFN in the culture medium, indicating that in the current work, the fundamental site of action of both NAC and GSH was restricted to inside the cell. Upon cellular uptake, SFN can also react or bind with various proteins.^66^ Taken together, in this study, possible reactions between GSH or NAC with SFN are fundamentally inside the cell; thus, their cytoprotective actions should be attributable to SFN conjugation, intracellular GSH generation, and antioxidant properties.

The observation that GSH can accumulate in cultured human lens cells following supplementation of the medium is both interesting and important. Work on the rodent lens has shown that GSH uptake occurs and is likely to be regulated by the Na^+^-independent organic anion transporter 3.^67^ Although GSH export by human lens cells has been reported,^68^ no detailed study of GSH uptake mechanisms in human lens cells has been conducted to date. This important aspect of GSH regulation will be a topic of future investigations.

**Figure 16.**
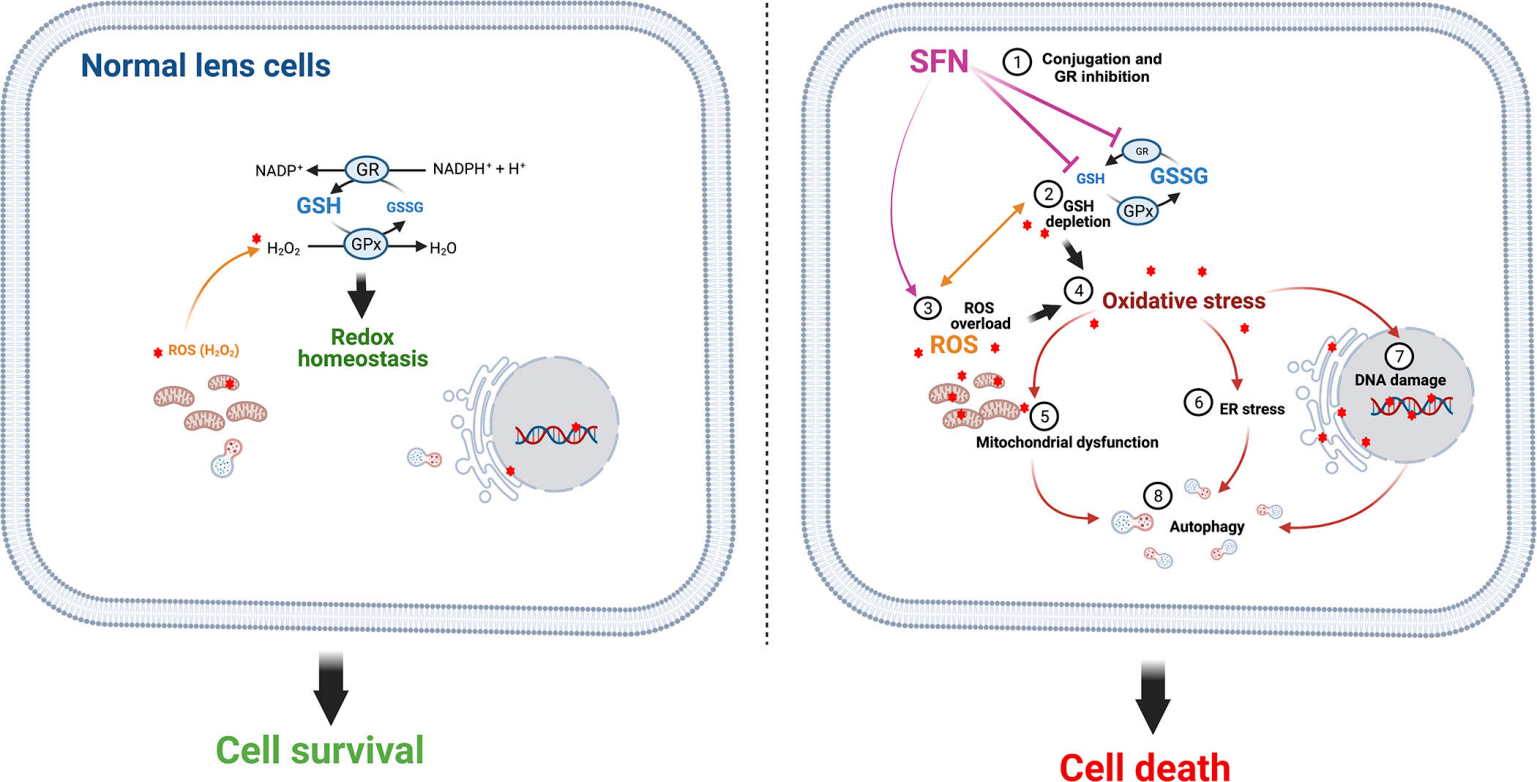
A proposed mechanism of SFN-induced cytotoxicity in human lens cells. Upon cellular entry, high supranutritional SFN concentrations rapidly deplete GSH via conjugation. The recovery of GSH levels is also impeded by the SFN-induced deficiency of its regeneration from GSSG by GR. Cells are quickly deprived of their major antioxidant defense and consequently become vulnerable to oxidative insults, even without additional stress stimuli; thus, the oxidative stress potential is elevated. SFN increases ROS levels, which is potentially a secondary effect of GSH depletion, and further exhausts the antioxidant defense, forming a vicious oxidative loop and leading to oxidative stress. Diverse cellular responses in mitochondria, the endoplasmic reticulum, and the nucleus are subsequently initiated to restore cellular homeostasis, but rather futilely given that the “life–death threshold” has been surpassed. As a result, these responses switch to their pro-apoptotic mode to drive cells to their grave.

ROS formation and GSH depletion are important in SFN-induced cytotoxicity, but which precedes which? A study done in human cancer cells showed that *o*-phenanthroline, an established antioxidant without affiliation with the GSH synthesis, and mitochondrial complex inhibitors could reduce ROS production and DNA damage caused by SFN but had no impact on the dramatic decrease in GSH levels.^42^ Nevertheless, whether GSH depletion acts causatively, additively, or synergistically with ROS generation remains to be elucidated. Considering the findings as a whole, we propose a possible model of the cytotoxicity of SFN in human lens cells (Fig. 16).

For the first time, to our knowledge, this study using a non-cancer human experimental system reports a complete model showing the interplay between redox status and physiological stress responses leading to SFN-induced cell death. GSH depletion and ROS formation are two key upstream mediators of oxidative stress-dependent cytotoxicity by SFN in both human lens epithelium and lens cells. Building on the fundamental work of Liu et al.^11^ indicating the therapeutic use of SFN cytotoxicity to prevent PCO, our elucidation of its mechanism provides essential insight to significantly advance the pharmaceutical applications of SFN to manage this condition. There should be no risk associated with SFN dietary supplements, regarding cytotoxicity, because intake of 200-µM SFN in humans has been reported to result in SFN plasma levels in the low micromolar range, less than 1 µM;^4^^,^^69^ this is well below the cytotoxic levels observed in the present and previously reported studies. Furthermore, dietary intake is not the potential delivery route of SFN for this application, but the use of a controlled drug delivery, such as the perfect capsule device, during cataract surgery is.^70^^,^^71^ Beyond the ocular system, the derived knowledge from this non-cancer study can also benefit other fields such as autoimmune disorders and hematological disorders, where SFN could be used to treat abnormal cell growth.^72^ Finally, this study highlights SFN as a promising agent to investigate a wide range of pathological conditions and aging associated with GSH imbalance.^73^

## Supplementary Material

Supplement 1
