# Supplementary material for: GSH Levels Serve As a Biological Redox Switch Regulating Sulforaphane-Induced Cell Fate in Human Lens Cells
Source: Invest Ophthalmol Vis Sci. 2021 Dec 2;62(15):2. doi: 10.1167/iovs.62.15.2 (PMC8648057; doi:10.1167/iovs.62.15.2)
Supplement: Supplement 1 [file iovs-62-15-2_s001.pdf]

Supplemental Figure S1

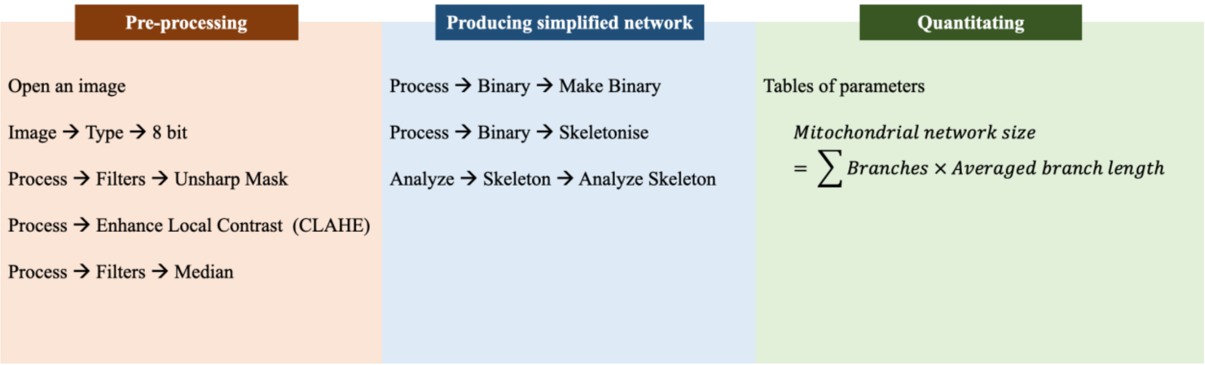

Figure S1.1: The workflow of mitochondrial network quantification. Images are pre-processed to improve quality prior to binarizing and skeletonizing. Descriptive parameters are calculated from the skeletonized image.

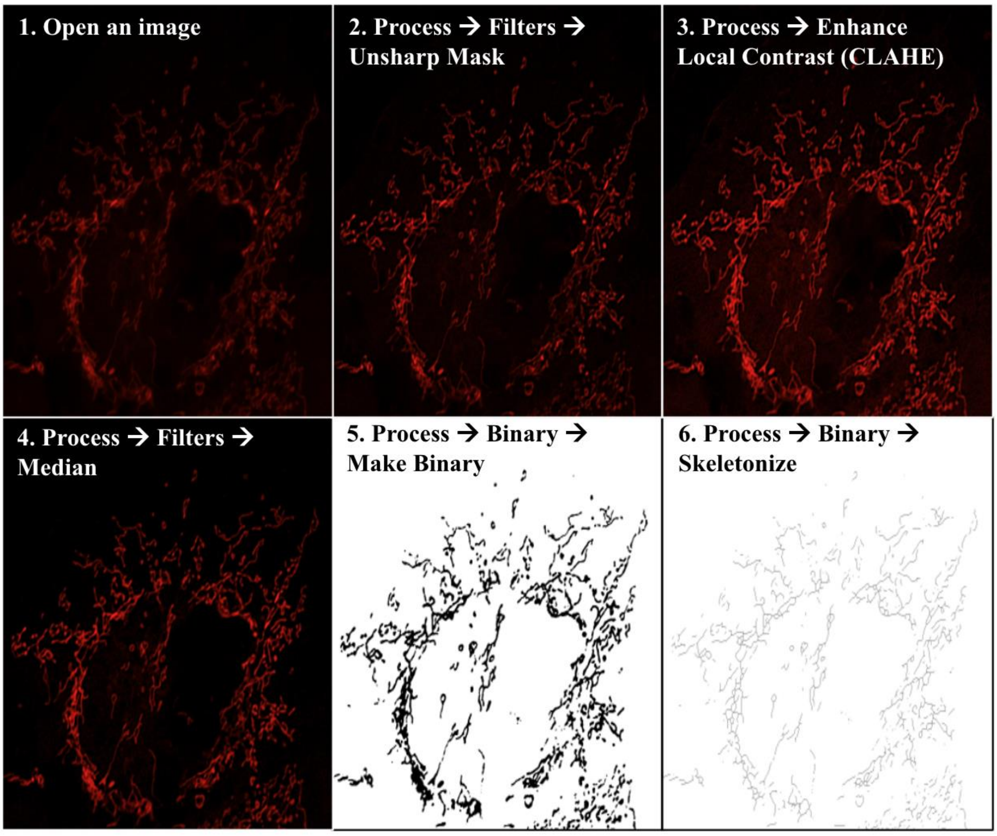

Figure S1.2: The representative preparation of an image. The image was pre-processed from step 2 – 4, and then made binary in step 5 and skeletonised in step 6. The skeleton was ready for quantitation.

Supplemental Figure S2

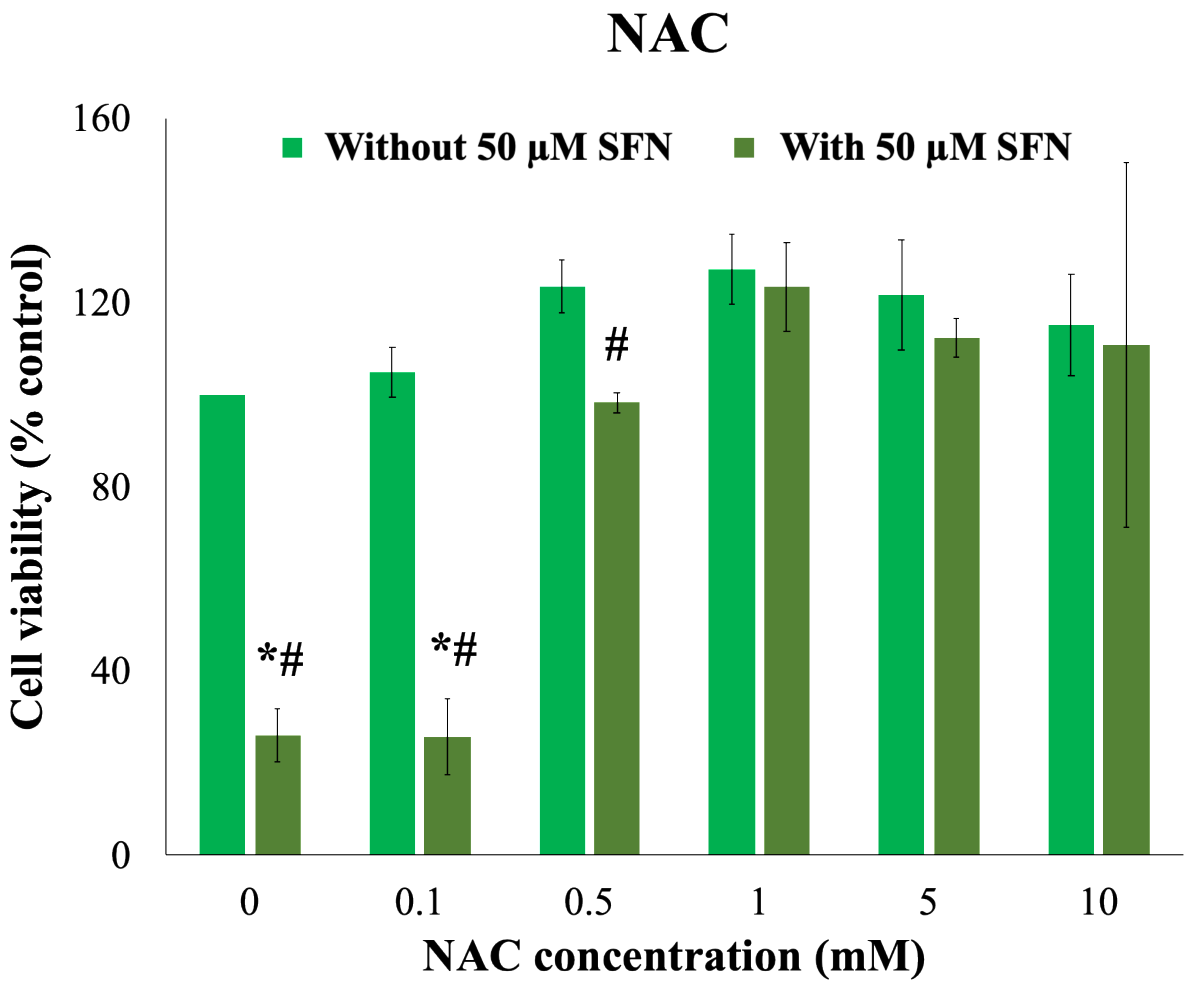

Figure S2.1: Effects of different concentrations of NAC on cell viability following SFN treatment in FHL124 cells. FHL124 cells were either maintained in serum-free (control) EMEM or EMEM supplemented with different concentrations (0.1 – 10 mM) of NAC . Following a period of 1 hour, half the preparations were treated with SFN (final concentration 50 µM) while the other half did not receive SFN treatment. Cell viability was measured after 18 hours treated with SFN using a Cell-TitreGlo Luminescent assay. Quantitative data are shown as mean ± SEM (n=3). An asterisk indicates a significant difference between the treated group and the untreated group ($p \leq 0.05$; ANOVA with Dunnett's post hoc test), a hashtag indicates a significant difference between with and without SFN groups of the same concentration of NAC ($p \leq 0.05$; independent Student's *t-test*).

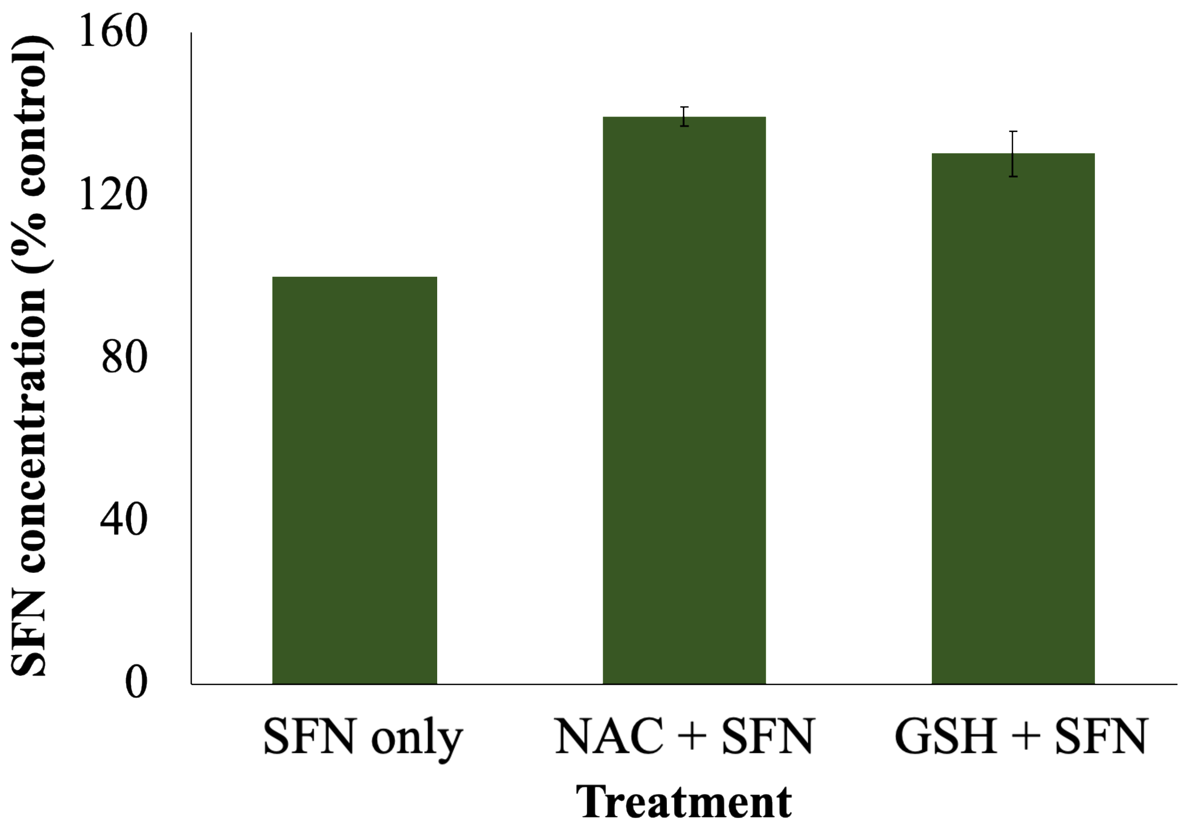

Figure S2.2: SFN concentration measured in the cell culture medium using LC-MS/MS. Culture medium (without cells or tissues) was either maintained in serum-free (control) EMEM or EMEM supplemented with NAC or GSH (final concentration of 1 mM). Following a period of 1 hour, SFN was added into culture medium samples (final concentration of 50 µM. After 1 hour, culture medium was sampled and analysed for SFN concentration using LC-MS/MS. Data are shown as mean ± SEM (n=3).

Supplemental Figure S3

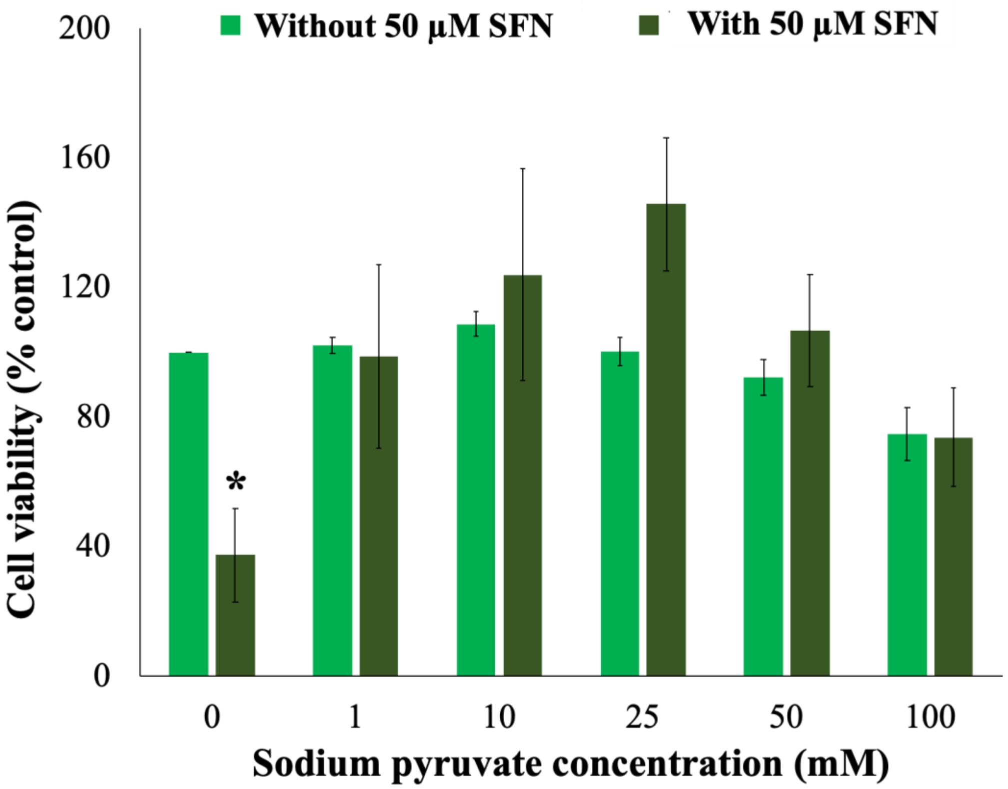

Figure S3.1: Effects of different concentrations of sodium pyruvate on cell viability following SFN treatment in FHL124 cells. FHL124 cells were either maintained in serum-free (control) EMEM or EMEM supplemented with different concentrations (1 – 100 mM) of sodium pyruvate . Following a period of 1 hour, half the preparations were treated with SFN (final concentration 50 µM) while the other half did not receive SFN treatment. Cell viability was measured after 18 hours treated with SFN using a Cell-TitreGlo Luminescent assay. Quantitative data are shown as mean ± SEM (n=4). An asterisk indicates a significant difference between the treated group and the untreated group ($p \leq 0.05$; ANOVA with Dunnett's post hoc test).

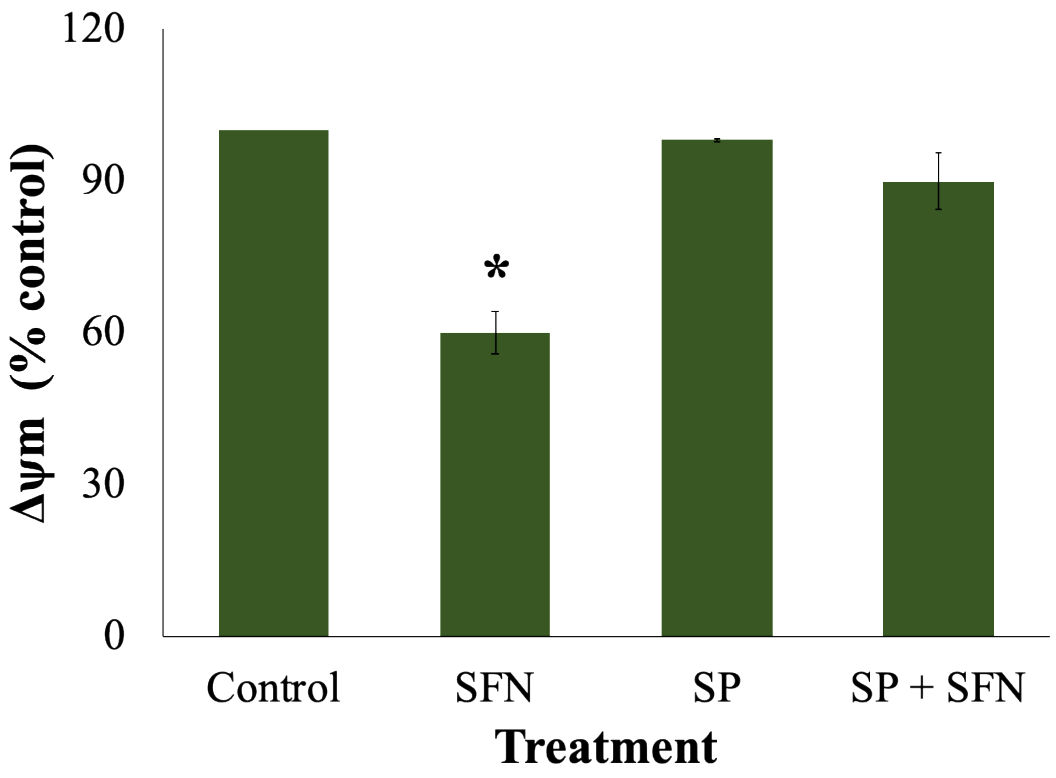

Figure S3.2: Sodium pyruvate (SP) protects cells from loss of mitochondrial membrane potential (Δψ_m) induced by SFN in FHL124 cells. FHL124 cells were either maintained in serum-free (control) EMEM or EMEM supplemented with 25 mM SP. Following a period of 1 hour, half the preparations were treated with SFN (final concentration 50 µM) while the other half did not receive SFN treatment. After 4 hours, Δψ_m was measured using TMRE signal at fluorescence excitation/emission wavelengths of 549/575 nm. Quantitative data are shown as mean ± SEM (n=3). An asterisk indicates a significant difference between the treated group and all other groups ($p \leq 0.05$; ANOVA with Tukey's post hoc test).

Supplemental Figure S4

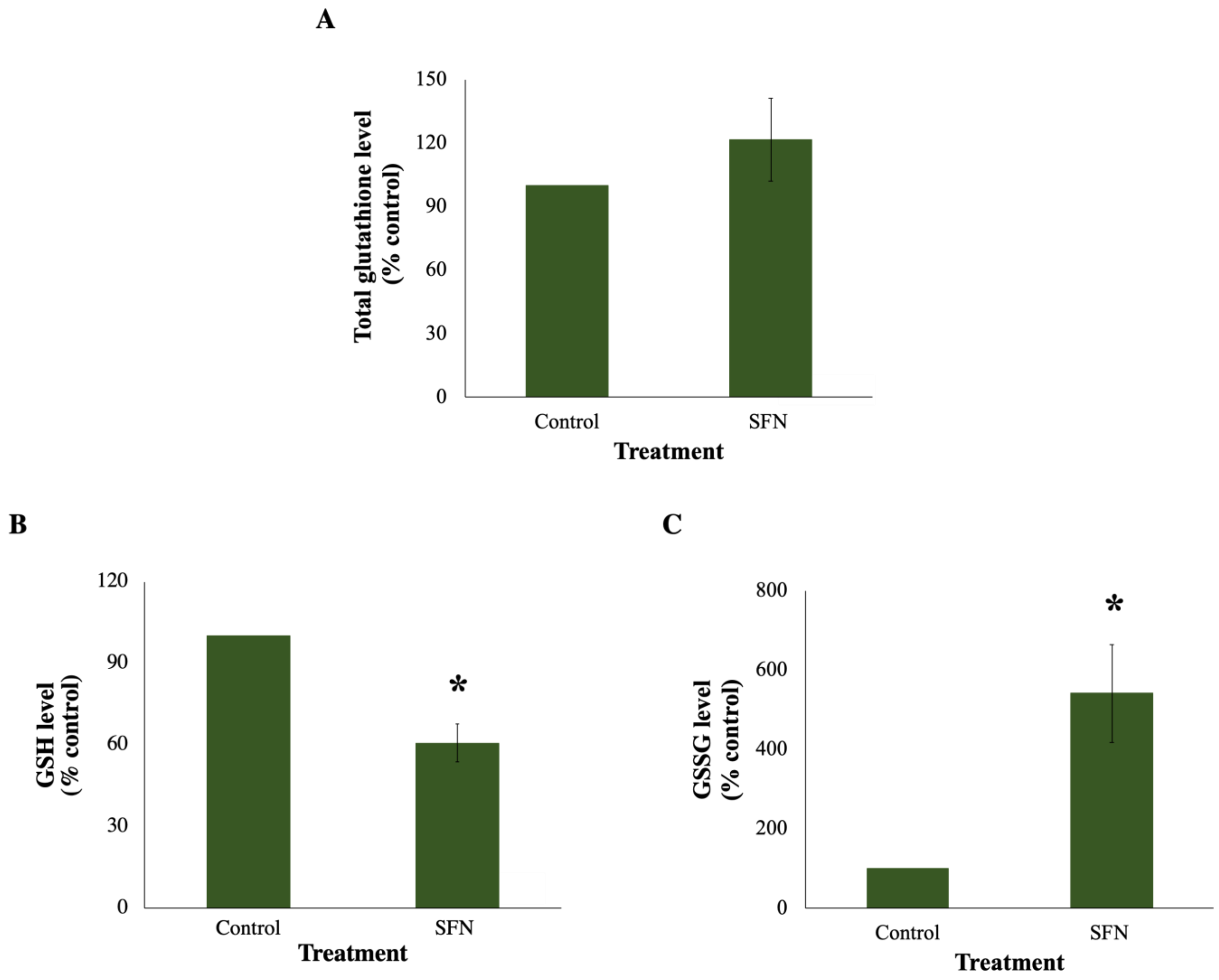

Figure S4: Impacts of different concentrations of SFN on total glutathione, GSH and GSSG levels in FHL124 cells. FHL124 cells were treated with different concentrations of SFN (0 – 50 μM) for 24 hours. Total glutathione and GSSG levels were measured using a GSH/GSSG luminescent assay. GSH levels were calculated based on the detected total glutathione and GSSG levels. A) Total glutathione, B) GSH and C) GSSG. Quantitative data are shown as mean ± SEM (n = 3). An asterisk indicates a significant difference between the treated group and the untreated control ($p \leq 0.05$; ANOVA with Dunnett's post hoc).

Supplemental Figure S5

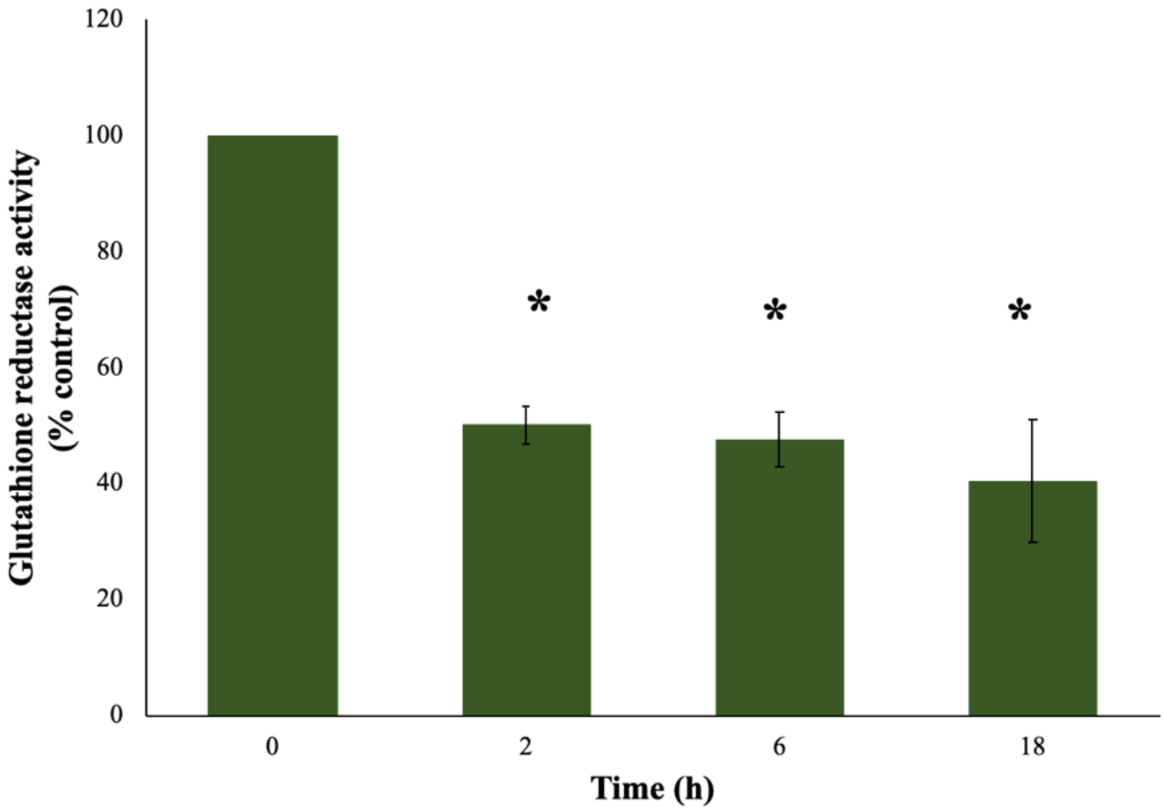

Figure S5: Activity of glutathione reductase in FHL124 cells treated with 50 µM SFN measured using a GR activity kit. Quantitative data are pooled from four separate experiments for FHL124 cells. Data are shown as mean ± SEM. Asterisk indicates a significant difference between the treated group and untreated controls ($p \leq 0.05$; ANOVA with Dunnett's post hoc test).

Supplemental Figure S6

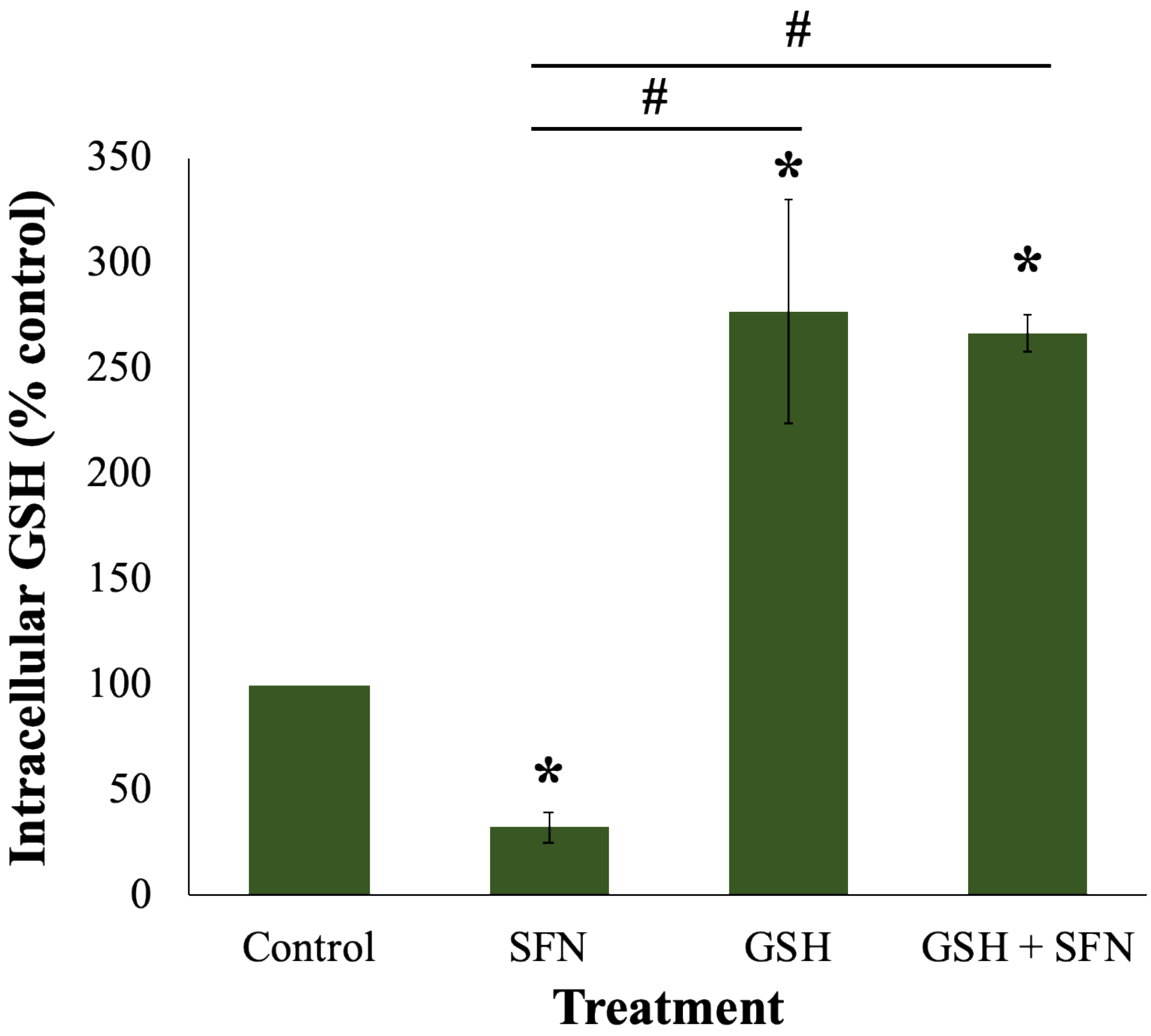

Figure S6: GSH supplementation increases intracellular GSH levels in FHL124 cells. FHL124 cells were either maintained in serum-free (control) EMEM or EMEM supplemented with 1 mM GSH. Following a period of 1 hour, half the preparations were treated with SFN (final concentration 50 µM) while the other half did not receive SFN treatment. After 1 hour, intracellular GSH were calculated based on the detected total glutathione and GSSG levels using a GSH/GSSG luminescent assay. Quantitative data are shown as mean ± SEM (n=3). An asterisk indicates a significant difference between the treated group and the control, and a hashtag indicates a significant difference between treated groups ($p \leq 0.05$; ANOVA with Tukey's post hoc test).
